# Plasminogen activation is required for the development of radiation-induced dermatitis

**DOI:** 10.1038/s41419-018-1106-8

**Published:** 2018-10-15

**Authors:** Mahsa Fallah, Yue Shen, Jessica Brodén, Assar Bäckman, Bertil Lundskog, Michael Johansson, Michael Blomquist, Kui Liu, Malgorzata Wilczynska, Tor Ny

**Affiliations:** 1 Department of Medical Biochemistry and Biophysics, Umeå University, 901-87 Umeå, Sweden; 2 Omnio AB, Tvistevägen 48, 907-36 Umeå, Sweden; 3 Department of Medical Biosciences, Pathology, Umeå University, 901-87 Umeå, Sweden; 4 Department of Radiation Sciences, Umeå University, 901-87 Umeå, Sweden

## Abstract

Skin damage caused by radiation therapy (radiodermatitis) is a severe side effect of radiotherapy in cancer patients, and there is currently a lack of effective strategies to prevent or treat such skin damage. In this work, we show with several lines of evidence that plasminogen, a pro-inflammatory factor, is key for the development of radiodermatitis. After skin irradiation in wild-type (*plg+/+*) mice, the plasminogen level increased in the irradiated area, leading to severe skin damage such as ulcer formation. However, plasminogen-deficient (*plg−/−)* mice and mice lacking plasminogen activators were mostly resistant to radiodermatitis. Moreover, treatment with a plasminogen inhibitor, tranexamic acid, decreased radiodermatitis in *plg+/+* mice and prevented radiodermatitis in *plg+/−* mice. Together with studies at the molecular level, we report that plasmin is required for the induction of inflammation after irradiation that leads to radiodermatitis, and we propose that inhibition of plasminogen activation can be a novel treatment strategy to reduce and prevent the occurrence of radiodermatitis in patients.

## Introduction

Approximately 50% of cancer patients receive some form of radiotherapy as a sole treatment or in combination with surgery or chemotherapy. Although radiotherapy techniques are continuously being developed, most patients still suffer from radiation-induced side effects which are mostly seen in tissues with rapidly proliferating cells such as the skin, gastrointestinal tract, and bone marrow^1^. In fact, the skin is affected to various degrees after any form of radiotherapy. The earliest visible skin reaction is erythema, which occurs in 90% of patients, and this can later evolve into desquamation or even into ulcers^2^. Radiation-induced dermatitis can be very painful and can severely affect the patient’s life quality^3^. The current strategies to treat radiation-induced dermatitis are suboptimal and include the application of dressings, antibiotics, and topical corticosteroids. Occasionally, severe wounds require skin grafting^4^.

The molecular mechanisms leading to radiation-induced dermatitis are not well understood, but DNA strand breaks and reactive oxygen species (ROS) induced by irradiation are considered to be the initial triggers for radiation-induced tissue damage^1,5^. These factors initiate cell death, especially in endothelial cells, fibroblasts, and keratinocytes. Radiation-induced damage to endothelial cells leads to the obstruction of the capillary lumen, ischemic damage, and vascular sclerosis;^3,6^ fibroblast dysfunction leads to defective collagen deposition and subsequent fibrosis; and damage to epithelial cells suppresses the formation of granulation tissue^3,7^. Signals sent by damaged cells and ROS induce the expression of transforming growth factor-β (TGF-β) and a burst of inflammation, both of which are known to be the main factors in the development of radiodermatitis^6,8^.

The pro-enzyme plasminogen, which is mainly produced in the liver and circulates in the blood at a relatively high concentration of 0.2 mg/ml^9^, is converted into the active protease plasmin by tissue-type plasminogen activator (tPA) or urokinase-type PA (uPA). Plasmin is a broad-spectrum protease that has an important role in fibrinolysis (the degradation of fibrin) and in the remodeling of the extracellular matrix^10,11^. Our previous studies have shown that plasminogen is a pro-inflammatory regulator that accumulates in wounds and accelerates wound healing^12,13^, but we have also shown that plasminogen plays a detrimental role in processes that involve excessive inflammation, such as sepsis^14^.

In the present study, we show that local irradiation of skin with ɣ-radiation in wild-type (*plg+/+*) mice induces plasminogen accumulation which is followed by the development of inflammation and subsequent skin damage. Notably, mice deficient in plasminogen (*plg−/−*) or mice lacking both plasminogen activators, PAs (*tPA−/−;uPA−/−*) are largely resistant to irradiation and do not develop radiodermatitis. Moreover, treatment with an inhibitor of plasminogen activation (tranexamic acid (TXA)) significantly delays the onset and decreases the severity of radiodermatitis in *plg+/+* mice and completely prevents radiodermatitis in *plg+/−* mice. Our study also for the first time links plasminogen activation to *TGF-β* expression in vivo, suggesting that inhibition of plasminogen can be used to suppress TGF-β activation for the prevention of radiodermatitis. Taken together, our data show that the inhibition of plasminogen activation during and immediately after radiotherapy might be a potential treatment strategy to protect cancer patients from radiodermatitis and possible other tissue damage.

## Materials and methods

### Animals

*Plg*-heterozygous (*plg+/−*) mice^15^ in a *C57BL/6* background were intercrossed to generate wild-type (*plg+/+*), heterozygous (*plg+/−*), and *plg*-deficient (*plg−/−*) mice. The mice were genotyped with a chromogenic assay^16^. Mice deficient in *tPA* or *uPA*^17^ were backcrossed for 10 generations with *C57BL/6* mice, and *tPA−/−* and *uPA−/−* mice were then intercrossed to generate the *tPA−/−;uPA−/−* mice. The genotypes of these mice were determined by PCR analysis^18^. Approximately 10- to 14-week-old mice were used for the experiments. The animals were kept under standard laboratory conditions, and the Regional Ethics Committee of Umeå University approved all of the experimental protocols.

### Radiation model

The dorsal skin of the mice was shaved 3 days prior to irradiation. For irradiation, the mice were anesthetized by intraperitoneal injection of 150 μl of a mixture containing 8% Ketaminol vet. (Intervet AB, Sollentuna, Sweden) and 5% Dormitor vet. (Orion Pharma AB, Espoo, Finland). The mice were placed inside a lead box with 2 cm thick walls to protect the whole body from radiation, and the dorsal skin was gently stretched out through a 4 cm gap at the bottom of the box and affixed with medical tape. The lead box with the mouse was then placed in a Gammacell 40 Exactor (Ashford, UK) that has two Cesium-137 sources. Radiation was given as a single dose of 1 Gy per min over 15 min (total dose of 15 Gy). The development of radiodermatitis was documented via digital photographs taken on different days after irradiation. The areas of the radiodermatitis lesions were quantified from the photographs using ImageJ (National Institute of Health, Bethesda, USA), and the severity of radiodermatitis was scored as 0 = no lesion, 1 = erythema, 2 = desquamation, and 3 = ulcer.

### Morphological and immunohistochemical analyses

Skin from the irradiated area was removed, fixed in 4% paraformaldehyde, and embedded in paraffin. Sections with a thickness of 6 µm perpendicular to the skin surface were prepared. The sections were deparaffinized with xylene and rehydrated through washes in a graded ethanol series. Antigen retrieval was performed using citrate buffer at 95 °C.

For histological analyses, the sections were stained with Mayer’s hematoxylin (Histolab, Gothenburg, Sweden) using a standard protocol. 8-Oxo-2′-deoxyguanosine was stained with goat polyclonal antibody (Abcam, Cambridge, UK) followed by donkey anti-goat IgG HRP (Abcam, Cambridge, UK) and DAB substrate (Vector Laboratories, Burlingame, USA) and the sections were counterstained with Mayer’s hematoxylin. Images were taken using a Leica DC300F digital camera attached to a Leica DM LB microscope (Leica, Wetzlar, Germany). Epidermal thickness was then measured (three measurements per skin sample) from the microscopy images using Adobe Photoshop. Quantification of area stained for 8-oxo-2′-deoxyguanosine was performed using ImageJ software.

Macrophages were stained with rabbit anti-mouse CD68 polyclonal antibody (Abcam, Cambridge, UK) followed by Dylight 594-labeled anti-rabbit IgG antibody (Vector Laboratories, Burlingame, USA). Neutrophils were stained with the rat anti-mouse Ly-6B.2 monoclonal antibody clone 7/4 (AbD Serotec, Oxford, UK) and followed by staining with biotinylated goat anti-rat IgG antibody (Santa Cruz Biotechnology, USA) and streptavidin-Alexa Fluor 647-conjugated antibody (Thermo Fisher Scientific, Waltham, USA). Nuclear extracellular traps (NETs) were stained with rabbit anti-citrullinated histone H3 antibody (Abcam, Cambridge, UK) followed by Dylight 488-labeled goat anti-rabbit IgG antibody (Vector Laboratories, Burlingame, USA). Apoptotic cells were detected via TUNEL (terminal deoxynucleotidyl transferase dUTP nick end labeling) assay (In Situ Cell Death Detection Kit, Roche, USA). Proliferative cells were stained with rabbit antibody against mouse Ki67 protein (Thermo Fisher Scientific, Waltham, USA) followed by Dylight 594-conjugated anti-rabbit IgG antibody (Vector Laboratories, Burlingame, USA). Fibrinogen/fibrin was stained with goat anti-mouse fibrinogen antibody (Nordic Immunological Lab, Tilburg, The Netherlands) followed by rabbit anti-goat Alexa Fluor 555-conjugated antibodies (Thermo Fisher Scientific, Waltham, USA). Vessels were stained with rat anti-mouse CD31 antibody (cluster of differentiation 31 protein) (BD Bioscience, San Jose, USA) followed by goat anti-rat IgG-TR antibody (Santa Cruz Biotechnology, Texas, USA). For fluorescence staining, 4′,6-diamidino-2-phenylindole (DAPI; Thermo Fisher Scientific, Waltham, USA) was used for staining nuclei, and images were captured with a Zeiss Axio Imager Z1 (Zeiss, Oberkochen, Germany). Quantification of fluorescent area was performed using ImageJ software.

### Analysis of skin extracts using ELISA

Dorsal skin samples from irradiated and non-irradiated mice were homogenized in lysis buffer (50 mM Tris-HCl buffer pH 8.0 with 120 mM NaCl, 1 mM EDTA, 6 mM EGTA, 1% NP-40, and 1 mM dithiothreitol) supplemented with PhosSTOP phosphatase inhibitors and cOmplete ULTRA mini protease inhibitor cocktail tablets (both from Roche, Basel, Switzerland). Skin samples were kept at −20 °C until use. The concentration of total protein in the extracts was quantified using a Pierce BCA protein assay kit according to the manufacturer’s instructions (Thermo Fisher Scientific, Waltham, USA). Mouse interleukin-6 (IL-6) and tumor necrosis factor-α (TNF-α) levels in the extracts were measured using specific enzyme-linked immunosorbent assay (ELISA) kits (eBioscience, San Diego, USA). Mouse plasminogen was quantified with a mouse plasminogen-specific ELISA kit (Omnio AB, Umeå, Sweden). IL-10 and IL-1β levels in skin extracts were measured using a mouse IL-10 ELISA kit (Abcam, Cambridge, UK) and a mouse IL-1β ELISA kit (Thermo Fisher Scientific, Waltham, USA) respectively.

### Western blot analysis

For quantification of phospho-Smad2 (PSmad2) in skin extracts, the protein extracts were separated by sodium dodecyl sulfate–polyacrylamide gel electrophoresis under reducing conditions using 4–12% gradient gels (NuPAGE, Thermo Fisher Scientific, Waltham, USA) and transferred to polyvinylidene difluoride membrane. PSmad2 (Ser465/467) was then detected with a rabbit monoclonal antibody (Cell Signaling Technology, Danvers, MA) followed by a goat anti-rabbit antibody coupled with peroxidase. Total Smad2 was detected with a rabbit monoclonal antibody (Cell Signaling Technology, Danvers, MA) and actin was detected with a monoclonal anti-actin antibody (Sigma-Aldrich, Saint Louis, USA). The protein bands were visualized with SuperBright ECL (Agrisera, Vännäs, Sweden) using ChemiDoc Touch Imaging System (Bio-Rad Laboratories, Hercules, USA). The protein band intensity was quantified using ImageJ software.

### Quantitative RT-PCR

Skin samples were homogenized in TRIzol (Ambion, Carlsbad CA, USA) using Precellys CK28R tubes on a Precellys 24 homogenizer (both from Bertin Technologies, Lyon, France) according to the manufacturer’s instructions. Total RNA was extracted with a PureLink RNA Mini Kit (Ambion, Carlsbad CA, USA) according to the manufacturer’s instruction. A 2.5 µg aliquot of total RNA was reverse-transcribed using a SuperScript VILO cDNA Synthesis Kit (Invitrogen, Carlsbad CA, USA) and diluted threefold with diethylpyrocarbonate-treated water. Gene expression was analyzed using quantitative real-time PCR (RT-PCR) with the comparative C_T_ method and with TATA-binding protein mRNA as the internal reference gene. The selection of the reference gene was based on data from RT² Profiler™ PCR Array Mouse Housekeeping Genes (Qiagen, Maryland, USA) and subsequent calculation of *M*-values using the geNorm software (Biogazelle NV, Gent, Belgium). The gene-specific primers and probes (TaqMan Gene Expression Assays) were obtained from Applied Biosystems (Foster City, USA), and 2× SsoAdvanced Universal Probes Supermix was from Bio-Rad (California, USA). Each sample was run in triplicate on the StepOnePlus Instrument (Applied Biosystems) using the real-time PCR conditions recommended for the 2× SsoAdvanced Universal Probes Supermix.

### Treatment with TXA

Because TXA (Meda AB, Stockholm, Sweden) is orally active, it was dissolved at 20 mg/ml in drinking water. The total daily dose was estimated at 100 mg/day. The treatment was initiated 2 days before irradiation and continued until day 10 after irradiation.

### Statistical analysis

**Fig. 1:**
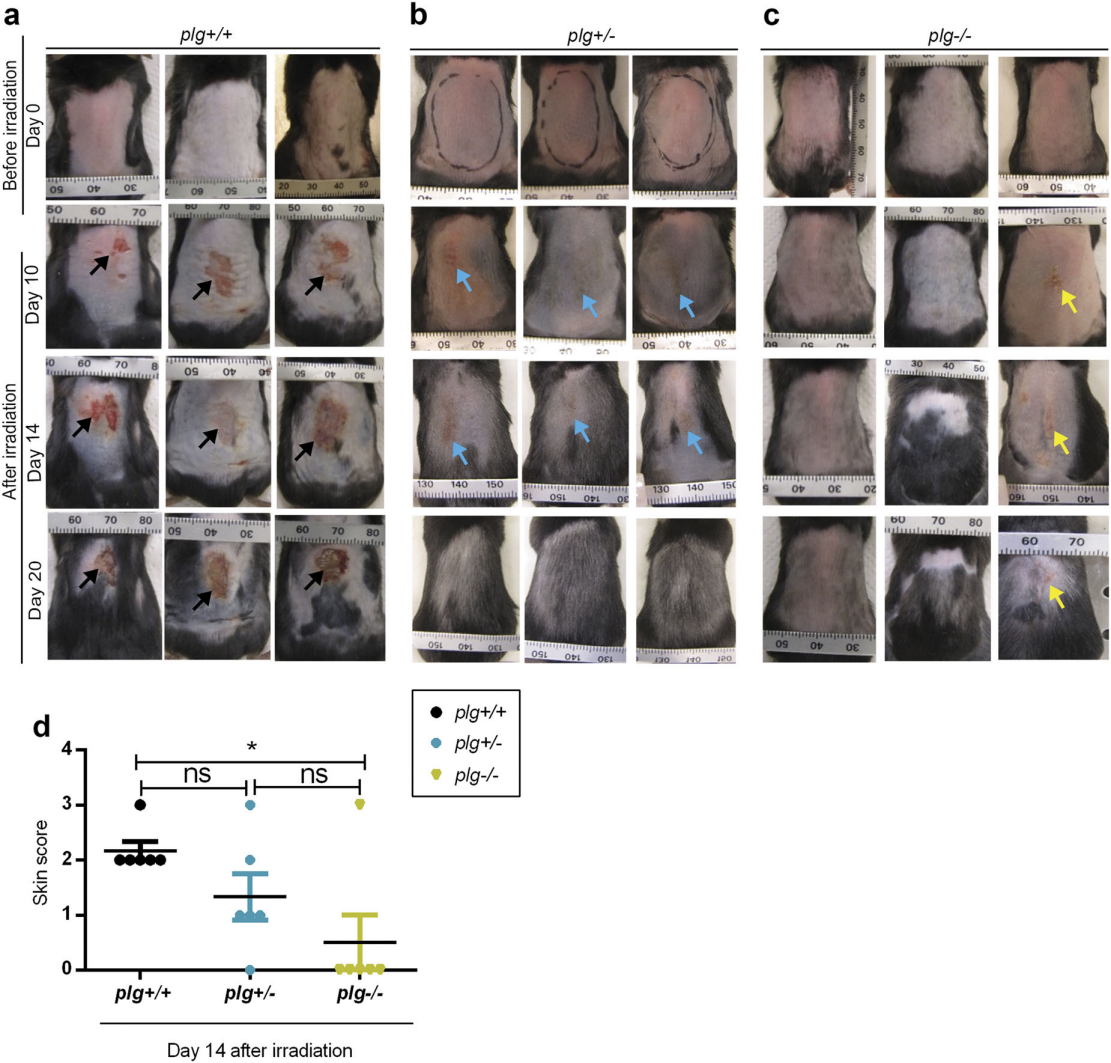
Plasmin(ogen) is a key factor for the development of radiodermatitis. Representative photographs of the dorsal skin of three different *plg+/+* (**a**), *plg+/−* (**b**), and *plg−/−* mice (**c**) at different time points after irradiation. Typical irradiated areas are marked on the photographs of the control *plg+/−* mice taken on day 0. Arrows show the wounds on the skin. **d** A comparison of the quality of dorsal skin in *plg+/+* (*n* = 6), *plg+/−* (*n* = 6), and *plg−/−* (*n* = 6) mice at day 14 after irradiation. The skin damage scores are 0 = normal skin, 1 = erythema, 2 = desquamation, and 3 = ulceration. The mean values are indicated (black lines) and SEM is shown. *P < 0.05

In Figs. 1d, 6b, d, and Supplementary Figure 1, the differences between two groups were analyzed with the Mann–Whitney *U*-test, and the results are expressed as scatter dot plots with the median indicated as a line. For the rest of the figures, the differences between two groups were analyzed with two-tailed *t*-tests and the differences between multiple groups were analyzed using one-way ANOVA, and the results are expressed as the mean ± SEM. *P* < 0.05 was defined as the significance threshold.

## Results

### Plasminogen deficiency in mice protects against radiation-induced dermatitis

Radiodermatitis is caused by the induction of excessive inflammation^8,19^. Because we previously showed that plasminogen is a pro-inflammatory regulator^12^, we tested the hypothesis that plasminogen is involved in the development of radiodermatitis. Dorsal skin of *plg+/+*, *plg+/−*, and *plg−/−* mice was exposed to a single ɣ-radiation dose of 15 Gy, and Fig. 1 shows representative photographs of the dorsal skin of the mice on different days after irradiation. Approximately 9 to 10 days after irradiation, all *plg+/+* mice developed erythema and then desquamation, and these wounds developed into ulcers between days 14 and 20 (Fig. 1a, d). The *plg+/−* mice, which have half the plasminogen level as that of *plg+/+* mice, developed erythema and mild desquamation around days 9 to 10, which healed before day 20 and never progressed to a severe form of radiodermatitis (Fig. 1b, d, and Supplementary Fig. 1). In contrast, most of the *plg−/−* mice showed no signs of dermatitis at any time point after irradiation (Fig. 1c, d), and only a few *plg−/−* mice developed wounds (Fig. 1c, d, and Supplementary Fig. 1). Of the 39 *plg−/−* mice used in all of our studies, only 21% developed any degree of radiodermatitis. In summary, the data suggest that the development of radiodermatitis is strongly related to plasminogen levels.

### Plasminogen is needed for the pathological changes associated with the development of radiodermatitis

**Fig. 2:**
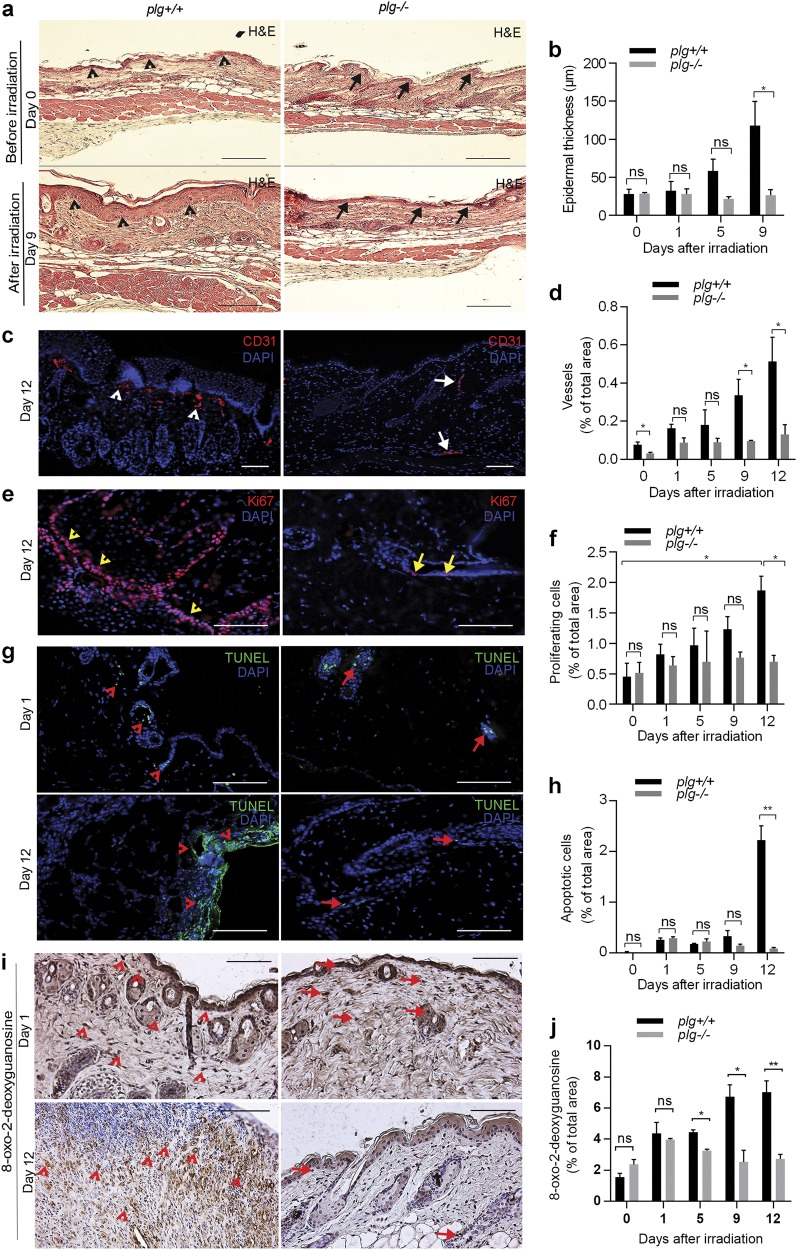
Plasminogen is needed for the histological changes in the skin after irradiation. **a** Representative images of hematoxylin and eosin (H&E)-stained skin sections from *plg+/+* and *plg−/−* mice before and on day 9 after irradiation. Arrowheads and arrows point to the epidermis in *plg+/+* and *plg−/−* mice, respectively. **b** The thickness of the epidermis in *plg+/+* and *plg−/−* mice on different days after irradiation measured in H&E-stained skin sections. **c** Representative images of skin sections stained for CD31 (red) from *plg+/+* and *plg−/−* mice on day 12 after irradiation. Arrowheads and arrows point to the blood vessels in *plg+/+* and *plg−/−* mice, respectively. **d** Quantification of blood vessels in *plg+/+* and *plg−/−* mice at different time points after irradiation. **e** Representative images of Ki67-stained (red) skin sections from *plg+/+* and *plg−/−* mice on day 12 after irradiation. Arrowheads and arrows point to the proliferating cells in *plg+/+* and *plg−/−* mice, respectively. **f** Quantification of proliferating cells in *plg+/+* and *plg−/−* mice at different time points after irradiation. **g** Representative images of TUNEL (green) staining of skin sections from *plg+/+* and *plg−/−* mice on day 1 and day 12 after irradiation. Arrowheads and arrows point to the apoptotic cells in *plg+/+* and *plg−/−* mice, respectively. **h** Quantification of apoptotic cells in *plg+/+* and *plg−/−* mice at different time points after irradiation. **i** Representative images of 8-oxo-2′-deoxyguanosine staining of skin sections from *plg+/+* and *plg−/−* mice on day 1 and day 12 after irradiation. Arrowheads and arrows point to cells with oxidized DNA in *plg+/+* and *plg−/−* mice, respectively. **j** Quantification of 8-oxo-2′-deoxyguanosine in *plg+/+* and *plg−/−* mice at different time points after irradiation. For all quantifications, *n* ≥ 3 per genotype. In all immunostained sections, DAPI (blue) was used for staining the nuclei. Scale bar = 100 µm. *P < 0.05; **P < 0.01.

As shown in Fig. 2a, b, and Supplementary Fig. 2, the thickness of the skin in *plg+/+* mice increased gradually after irradiation, and at day 9 when erythema was observed the skin was approximately 4.6-fold thicker compared to non-irradiated skin (Fig. 2a, b). In sharp contrast, the thickness of the epidermis in *plg−/−* mice was not significantly different at any time point after irradiation (Fig. 2a, b). Moreover, thickening of the skin in *plg+/+* mice suffering from radiodermatitis was accompanied by a significantly elevated number of blood vessels (shown by staining for CD31), which was not the case in *plg−/−* mice (Fig. 2c, d, and Supplementary Fig. 3).

In agreement with the increased thickness of the epidermis, the number of proliferating cells in *plg+/+* mice (as indicated by Ki67 staining) also gradually increased, with the highest rate of cell proliferation at day 12 after irradiation and coinciding with ulcer formation (Fig. 2e, f, and Supplementary Fig. 4). In contrast, the number of proliferating cells did not significantly increase in the irradiated *plg−/−* mice (Fig. 2e, f, and Supplementary Fig. 4).

Irradiation causes apoptosis due to the creation of DNA strand breaks^20^. Similar rates of apoptosis were observed in both *plg+/+* and *plg−/−* mice at day 1 after irradiation, indicating that plasminogen has no effect on apoptosis at early time points after irradiation. However, at day 12 the number of apoptotic cells had increased by 8.5-fold in the *plg+/+* mice, while the number of apoptotic cells had slightly decreased in the *plg−/−* mice compared to day 1 (Fig. 2g, h, and Supplementary Fig. 5). The high levels of apoptosis in *plg+/+* mice on day 12 coincided with the development of radiodermatitis, and the lack of elevated apoptosis in *plg−/−* mice indicates that the development of radiodermatitis is suppressed in these mice.

Radiotherapy induces oxidative stress via induction of high levels of ROS. ROS are responsible for oxidation of cellular proteins and DNA, and are one of the major factors leading to cellular damage after irradiation^21^. To detect the level of oxidative stress in irradiated skin, we stained skin sections for 8-oxo-2′-deoxyguanosine that correlates with the damage of DNA caused by ROS^22^. As shown in Fig. 2i, j, and Supplementary Fig. 6, the same levels of DNA damage were detected in *plg+/+* and *plg−/−* mice at day 1 after irradiation. At the later time points, the DNA damage increased significantly in *plg+/+* mice, while it declined to normal levels in *plg−/−* mice, indicating that plasminogen deficiency preventes the formation of excessive oxidative stress in the later time points after irradiation.

Taken together, the above data show that in the absence of plasminogen, irradiation does not induce any of the visible pathological events that normally occur during the development of radiodermatitis.

### Neutrophils and macrophages infiltrate the irradiated skin of *plg+/+* mice but not that of *plg−/−* mice

**Fig. 3:**
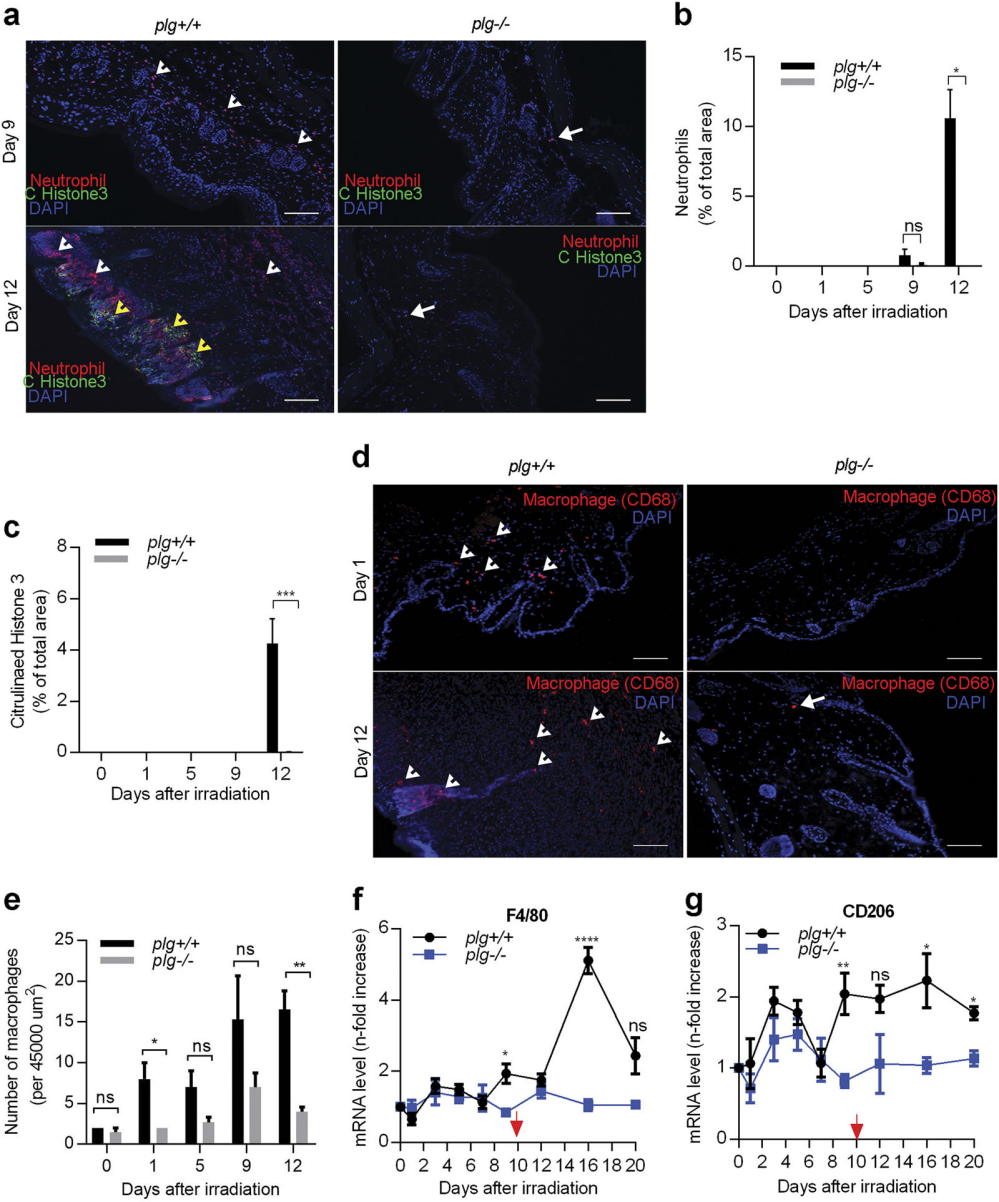
Large numbers of inflammatory cells infiltrate the irradiated skin of *plg+/+* but not *plg−/−* mice. **a** Representative photographs of skin sections from *plg+/+* and *plg−/−* mice on days 9 and 12 after irradiation stained for neutrophils (red), DAPI (blue), and NETs (citrullinated histone H3) (green). White arrowheads and arrows point to neutrophils in *plg+/+* and *plg−/−* mice, respectively. Yellow arrowheads point to NETs in *plg+/+* mice. **b**, **c** Quantification of neutrophils and NETs in the skin sections from *plg+/+* and *plg−/−* mice on different days after irradiation (*n* ≥ 3 per genotype). **d** Representative immunostaining for macrophages (red) in sections from irradiated *plg+/+* and *plg−/−* mouse skin on days 1 and 12 after irradiation. Arrowheads and arrows point to macrophages in *plg+/+* and *plg−/−* mice, respectively. **e** Quantification of macrophages in the skin sections from plg*+/+* and *plg−/−* mice on different days after irradiation (*n* ≥ 3 per genotype). Scale bar = 100 µm. **f**, **g** Expression of *F4/80* and *CD206* mRNA, respectively, in skin extracts from *plg*+/+ and *plg−/−* mice on different days after irradiation quantified by RT-PCR (*n* ≥ 4 per genotype). The red arrow indicates the day when dermatitis becomes visible in *plg*+/+ mice. *P < 0.05; **P < 0.01; ***P < 0.005; ****P < 0.001

To determine if the lack of radiodermatitis in *plg−/−* mice is due to suppressed inflammatory cell infiltration after irradiation, we stained skin sections of both genotypes for neutrophils. We found no detectable neutrophils from day 0 to day 5 post irradiation in either genotype (Supplementary Fig. 7). In *plg+/+* mice, neutrophils began to infiltrate the skin on day 9 post irradiation, and they reached very high numbers on day 12, which correlated with the development of ulcers (Fig. 3a, b). However, in *plg−/−* mice, almost no neutrophils were detected in the skin at any time point after irradiation (Fig. 3a, b). It is known that during inflammation neutrophils can release NETs (de-condensed chromatin linked with various cytotoxic proteins) that can cause tissue damage^23^. At day 12 post irradiation, no NETs were observed in the irradiated skin of *plg−/−* mice, which was in sharp contrast with the large numbers of NETs in the *plg+/+* mice that appeared on day 12 when radiodermatitis developed (Fig. 3a, c, and Supplementary Fig. 7). These data suggest that plasminogen/plasmin is an indispensable trigger for the development of inflammation after irradiation. Without plasminogen, inflammation does not develop.

It is well known that activated macrophages migrate to irradiated tissues^24^. As shown in Fig. 3d–f, in *plg+/+* mice, the number of macrophages started to increase by days 1–5 post irradiation and continued to increase during following days which coincides with the development of erythema and ulcers. In contrast, the number of macrophages in *plg−/−* mice only increased slightly around days 5–9 and then returned to a low basal level after that (Fig. 3d–f and Supplementary Fig. 8). These data suggest that in *plg+/+* mice the infiltration of macrophages occurred in two phases—the first phase was just after irradiation, and the second phase occurred at the time when erythema had developed and progressed into ulcers. In *plg−/−* mice, it is apparent that macrophage infiltration occurred only early after irradiation because these mice did not subsequently develop radiodermatitis. To test phagocytic ability of the macrophages, we measured the expression of CD206 (mannose receptor C type 1). CD206 expression was low in pro-inflammatory M1 macrophages, but high in anti-inflammatory M2 macrophages^25^. As shown in Fig. 3g, the expression of CD206 increased from days 3 to 5 in both *plg+/+* and *plg−/−* mice, but from day 7 onward the expression of CD206 increased further and remained high throughout the development of radiodermatitis in *plg+/+* mice, while it returned to low basal levels in *plg−/−* mice. This indicates a higher phagocytic activity in *plg+/+* mice as compared to *plg−/−* mice that most likely is linked with an extended tissue damage in the *plg+/+* mice. In addition, a fivefold increase of F4/80 expression at day 16 in *plg+/+* mice was accompanied by only about a twofold increase of CD206 expression, suggesting that the number of pro-inflammatory M1 macrophages increased in radiation-induced wounds at day 16 (Fig. 3f, g).

### Plasminogen accumulates in irradiated skin and induces the factors causing radiation-induced tissue damage

**Fig. 4:**
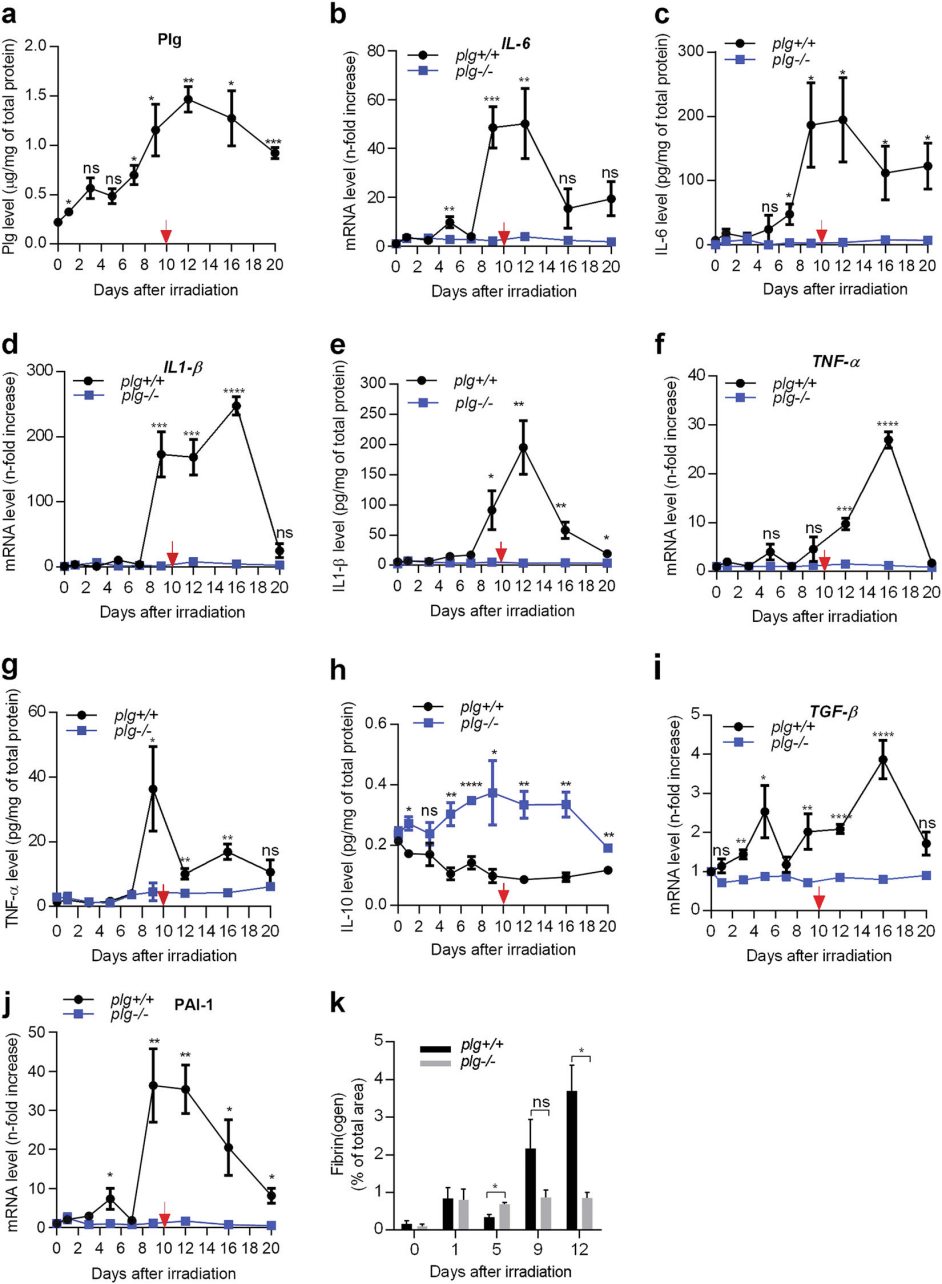
Plasminogen accumulates in the skin after irradiation and induces factors that are involved in post-irradiation tissue damage. **a**–**j** Skin samples were taken from *plg*+/+ and *plg−/−* mice on different days after irradiation (*n* ≥ 4 per time point), and specific mRNA and protein levels were measured using RT-PCR and protein-specific ELISA, respectively. **a** For statistical significance, levels of plasminogen in the skin of *plg+/+* mice at different time points was compared to day 0. **b**, **c** IL-6 mRNA and protein levels. **d**, **e** IL-1-β mRNA and protein levels. **f**, **g** TNF-α mRNA and protein levels. **h** Protein levels of IL-10. **i**, **j** *TGF-β* and *PAI-1* mRNA levels. **k** Fibrin(ogen) levels in skin sections of *plg+/+* and *plg−/−* mice at different time points after irradiation (*n* ≥ 3 per genotype). *P < 0.05; **P < 0.01; ***P < 0.005; ****P < 0.001

We have shown previously that plasminogen is transported to wounded skin by immune cells where its levels increase by approximately sevenfold^12^. Here, we have shown that the level of plasminogen in the irradiated *plg+/+* mice increased gradually from day 1 after irradiation and reached the highest level (an approximately sevenfold increase) from days 9 to 12, after which it began to decrease (Fig. 4a).

The development of acute radiodermatitis is correlated with high levels of inflammatory cytokines^24^. As shown in Fig. 4b, c, the expression and protein level of *IL-6* in *plg+/+* mice began to increase from day 5 and reached the highest level from days 9 to 12 post irradiation when radiodermatitis developed. The expression and protein levels of *IL1-β* and *TNF-α* in these mice were low until day 7 post irradiation, but increased dramatically on day 9 (Fig. 4d–g). In contrast, in *plg−/−* mice these cytokines remained at basal levels at all time points after irradiation. The only exception was IL-10 for which protein levels were significantly higher in *plg−/−* mice during all time points after irradiation, compared to the *plg+/+* mice. IL-10 is an anti-inflammatory cytokine which downregulates the production of pro-inflammatory cytokines after radiation exposure^26^. The relatively high levels of IL-10 in *plg−/−* mice may be, at least partially, responsible for the lack of inflammatory burst in irradiated skin in these mice. Since mRNA levels for IL-10 do not correlate with IL-10 protein levels^27^, the expression of mRNA for IL-10 is not shown here.

It is worth noting that TGF-β is one of the most important factors that contribute to tissue injury after irradiation^19^. As shown in Fig. 4i, the expression of *TGF-β* mRNA in the irradiated skin of *plg+/+* mice occurred in two phases and followed the macrophage accumulation. The first peak of *TGF-β* expression was observed around day 5 (a 2.5-fold increase), and the second peak was seen from days 9 to day 16 (an approximately fourfold increase). In sharp contrast, no increase in *TGF-β* expression was detected in the irradiated skin of *plg−/−* mice, showing that in the absence of plasminogen TGF-β was not induced and thus could not initiate the inflammation that is required for radiodermatitis to develop. These data suggest that plasminogen may be involved in activation of the TGF-β signaling pathway. To support this conclusion, we have quantified phosho-Smad2 in extracts from irradiated skin by western blot. As shown in Supplementary Fig. 9, phosphorylation of Smad2 was increased in *plg+/+* mice at days 9–16, whereas it was very low in *plg−/−* mice at these time points.

PAI-1 (*Serpine1*, plasminogen activator inhibitor type 1) is one of the molecules that are regulated by TGF-β^28,29^. In addition to being an inhibitor of plasminogen activators, PAI-1 can also regulate cell migration through a mechanism that is not dependent on its inhibitory activity^30^. TGF-β can induce the expression of PAI-1 via the Smad pathway, and high levels of PAI-1 have been implicated in intestinal radiation injury^29,31^. Here we found that the pattern of PAI-1 expression in the irradiated skin followed the expression of *TGF-β*. In *plg+/+* mice, PAI-1 expression peaked first on day 5 (a 5-fold increase) and then from days 9 to 12 (an approximately 36-fold increase). In *plg−/−* mice, however, PAI-1 expression remained low at all time points after irradiation (Fig. 4j). This suggests that PAI-1 might also be involved in radiation-induced skin damage.

Irradiation and inflammation result in increased blood vessel permeability and leakage of plasma proteins into tissues^32^. Fibrin deposits appeared on day 1 after irradiation to the same extent in both *plg+/+* and *plg−/−* mice. In *plg−/−* mice, this level remained stable over all subsequent days. However, in *plg+/+* mice the fibrin levels decreased significantly on day 5 after irradiation, but increased again from days 9 to 12 concomitant with inflammation and ulcer formation (Fig. 4k, and Supplementary Fig. 10).

### Plasminogen activation is necessary to induce inflammation and the expression of molecules that are involved in radiation-induced tissue damage

**Fig. 5:**
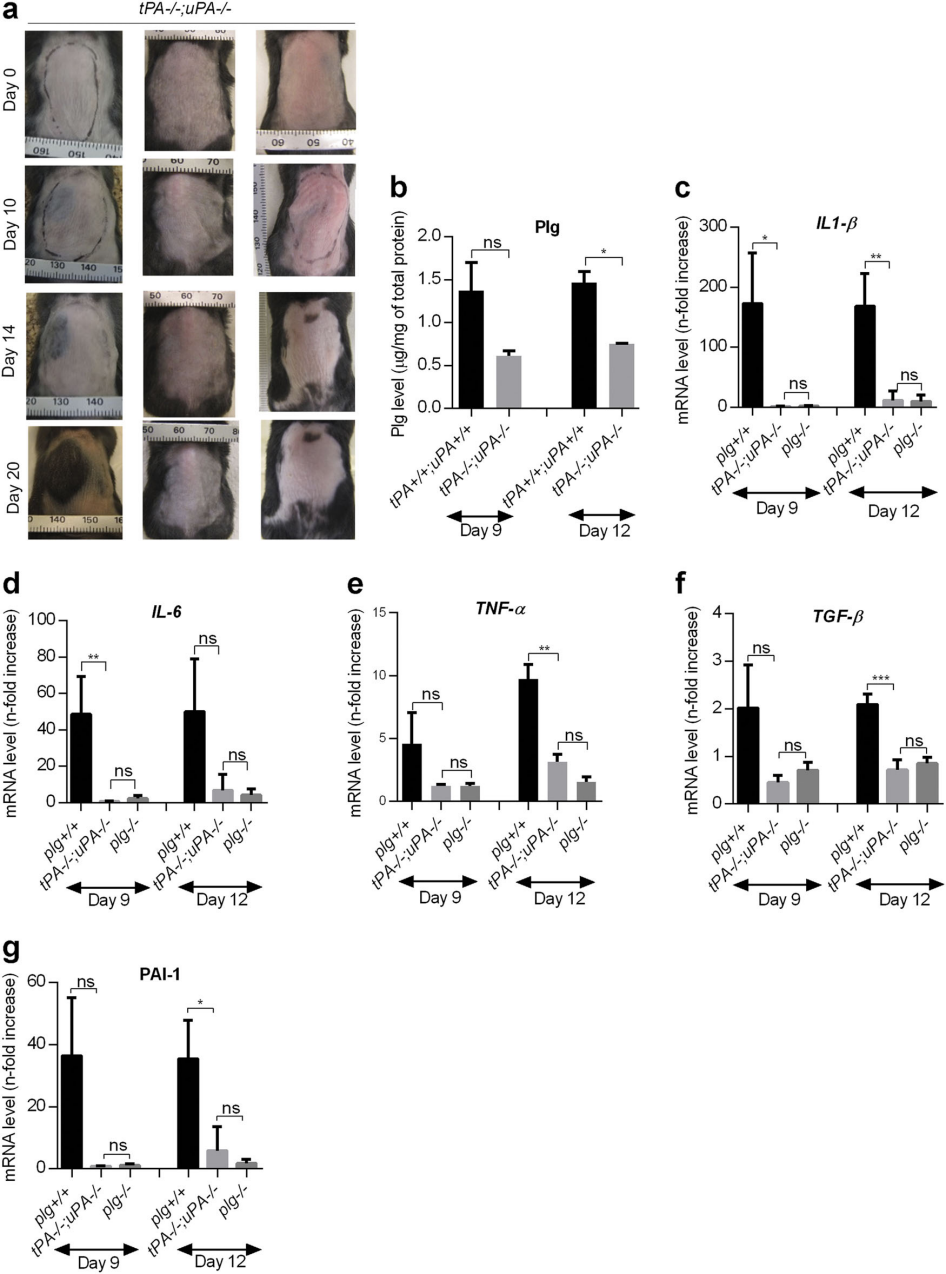
Plasmin activity is necessary for the development of radiodermatitis. **a** Representative photographs of the dorsal skin of the *tPA−/−;uPA−/−* mice at different time points after irradiation. **b** Plasminogen levels in skin extracts from *tPA+/+;uPA+/+* mice and *tPA−/−;uPA−/−* mice after irradiation measured by ELISA. **c–g** RT-PCR measurement of mRNA levels of *IL1-β* (**c**), *IL-6* (**d**), *TNFα* (**e**), *TGF-β* (**f**), and *PAI-1* (**g**) in irradiated skin of *plg+/+*, *plg−/−*, and *tPA−/−;uPA−/−* mice. *P < 0.05; **P < 0.01; ***P < 0.005

The above results indicate that plasminogen is required for the development of radiodermatitis. To confirm that plasminogen activation is important for this process, we used the *tPA−/−;uPA−/−* mice that have normal plasminogen levels but plasminogen cannot be activated to plasmin. Irradiated *tPA−/−;uPA−/−* mice did not develop any form of radiodermatitis at any time point after irradiation (Fig. 5a), and plasminogen levels in the irradiated skin of these mice were significantly lower than in irradiated *tPA+/+;uPA+/+* mice (Fig. 5b), indicating that the total plasminogen accumulation partially depends on the activation of plasminogen into plasmin. However, the mRNA and protein levels of pro-inflammatory cytokines (*IL1-β*, *IL-6*, and *TNF-α*), protein levels of the anti-inflammatory cytokine IL-10, and the mRNA levels of *TGF-β* and *PAI-1* in the irradiated skin from the *tPA−/−;uPA−/−* mice were all comparable to the levels in the *plg−/−* mice (Fig. 5c–g and Supplementary Fig. 11). Taken together, these data strongly suggest that plasmin, the active form of plasminogen, is responsible for inducing key factors that are involved in the development of radiodermatitis.

### Inhibition of plasminogen activation by TXA delays the onset and decreases the severity of radiodermatitis in *plg+/+* mice and prevents radiodermatitis in *plg+/−* mice

**Fig. 6:**
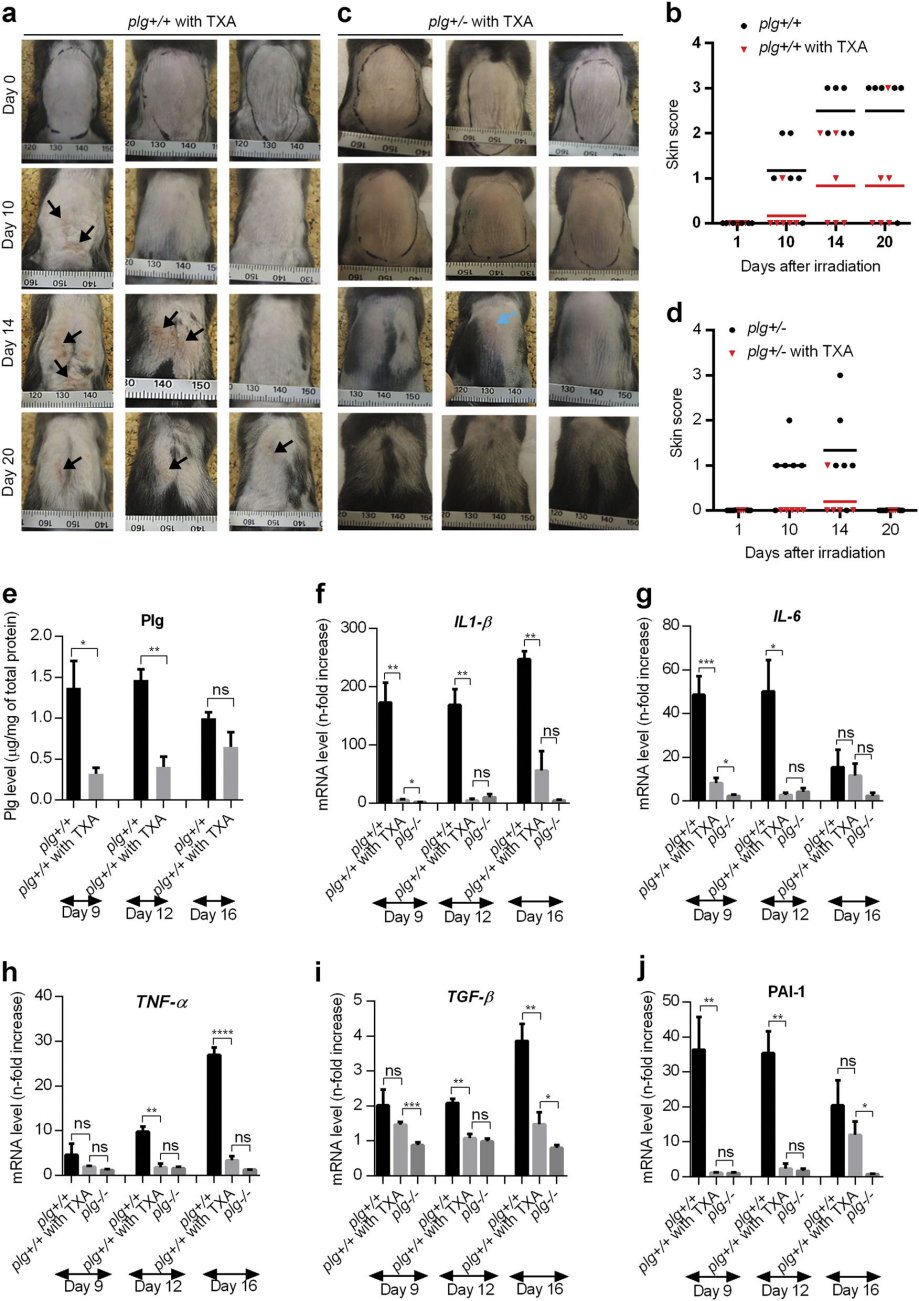
Tranexamic acid inhibits the development of radiodermatitis in *plg+/+* and *plg+/−* mice. *plg+/+* and *plg+/−* mice were treated with TXA in their drinking water from 2 days before irradiation until day 10 post irradiation. Control mice were irradiated but did not receive TXA. **a**, **b** Representative photographs of the dorsal skin of irradiated *plg+/+* mice treated with TXA, and a comparison of the quality of the dorsal skin in irradiated control and TXA-treated *plg+/+* mice (*n* = 6 per time point). **c**, **d** Representative photographs of the dorsal skin of irradiated *plg+/−* mice treated with TXA, and a comparison of the quality of the dorsal skin in irradiated control and TXA-treated *plg+/−* mice (*n* = 6 per time point). Arrows show the wounded skin area. **e** Plasminogen levels in the skin extracts of control and TXA-treated *plg+/+* mice on different days after irradiation. **f**–**j** RT-PCR measurement of mRNA levels of *IL1-β* (**f**), *IL-6* (**g**), *TNF-α* (**h**), *TGF-β* (**i**), and *PAI-1* (**j**) in the irradiated skin of control and TXA-treated *plg+/+* and in *plg−/−* mice on different days after irradiation. *P < 0.05; **P < 0.01; ***P < 0.005; ****P < 0.001.

Plasminogen binds to lysine residues on fibrin and on plasminogen-specific cell receptors, and it is then activated into plasmin by the PAs^33^. Lysine analogs, such as TXA, inhibit plasminogen activation and are used in the clinic to treat clotting disorders^34^. In this study, we added TXA to the drinking water of *plg+/+* and *plg+/−* mice starting from 2 days before irradiation and ending on day 10 post irradiation. *Plg+/+* mice treated with TXA showed delayed onset and reduced severity of radiodermatitis (Fig. 6a, c) compared to the irradiated *plg+/+* mice without TXA. Moreover, in *plg+/−* mice treated with TXA the development of radiodermatitis was largely inhibited, and only low-grade erythema developed on day 14, which had healed by day 20 (Fig. 6b, d, and Supplementary Table 1).

As expected, treatment with TXA led to a reduced accumulation of plasminogen in irradiated skin. On day 9, the plasminogen level in the irradiated skin of TXA-treated *plg+/+* mice was approximately 4.5-fold lower than that of control irradiated *plg+/+* mice (Fig. 6e). Because the TXA treatment ended on day 10, the plasminogen level in TXA-treated mice began to slowly increase on day 16 post irradiation, but this increase did not seem to be sufficient to induce radiodermatitis. The mRNA and protein levels for pro-inflammatory cytokines (*IL1-β*, *IL-6*, and *TNF-α*) were all low in TXA-treated *plg+/+* mice and were comparable to those in irradiated *plg−/−* mice (Fig. 6f–h and Supplementary Fig. 12). Treatment of *plg+/+* mice with TXA also resulted in decreased expression of *TGF-β* and *PAI-1* (Fig. 6i, j). However, the low protein levels of the anti-inflammatory IL-10 observed in irradiated skin of *plg+/+* mice increased significantly after TXA treatment (Supplementary Fig. 12).

## Discussion

Because the molecular mechanisms behind the development of radiation-induced dermatitis are not fully understood, current treatments of radiation-induced wounds are still based on conventional wound treatments. Here, we provide compelling evidence indicating that plasminogen is a critical factor in the development of radiodermatitis. We found that the development of radiodermatitis in mice is initiated by the accumulation of plasminogen in the irradiated skin where it is activated to plasmin. We also showed that plasmin plays a critical role in recruiting neutrophils and macrophages and inducing the expression and activation of *TGF-β* and various inflammatory factors that are known to cause injury in healthy tissues. Finally, we found that genetic ablation of plasminogen or PAs prevents the formation of radiodermatitis in mice and that the inhibition of plasminogen activation by TXA significantly decreases the severity of radiodermatitis in *plg+/+* mice and prevents radiodermatitis in *pl*g+/− mice. Collectively, these results show that the expression of *TGF-β* and subsequent inflammatory reactions are not induced by irradiation in *plg*−/− mice and in mice in which plasminogen activation is prevented. This suggests that the inhibition of plasminogen/plasmin might be a novel treatment for preventing radiodermatitis in cancer therapy.

**Fig. 7:**
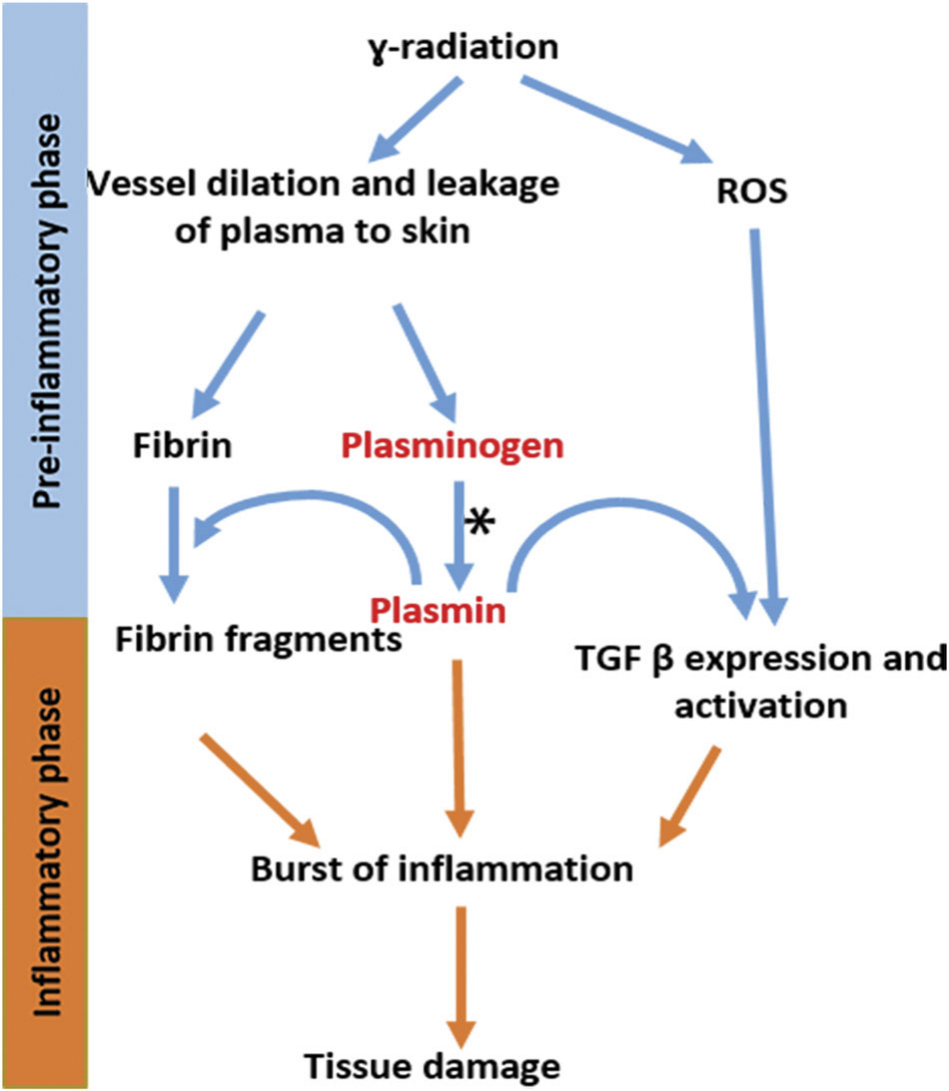
Proposed model for the role of plasminogen in the development of radiodermatitis. Inhibiting plasminogen activation by TXA (star) prevents the induction of the burst of inflammatory reaction

Based on our current findings and previously published data, we propose a new model for the role of plasmin in the development of skin damage after γ-irradiation (Fig. 7). Considering the timing of events, the interval between irradiation and the appearance of radiodermatitis can be divided into two phases. The first phase, which we call the “pre-inflammatory phase” (days 1 to 5 post irradiation), is characterized by a lack of inflammation. At the beginning of this phase, irradiation of the skin causes the production of ROS and induces vessel permeability^32^. Plasma diffuses from the vessels, transporting fibrinogen and plasminogen to the dermis. In the tissue environment, fibrinogen is rapidly converted to fibrin and binds to plasminogen^33^. Plasminogen then becomes activated to plasmin which degrades fibrin and produces various biologically active fibrin fragments^35,36^. Plasminogen also binds to cell-surface receptors where it is activated by cell-bound PAs^37^. Plasmin, together with ROS, activates latent TGF-β^38,39^ and the associated signaling pathways, and this leads to the enhanced expression of *TGF-β*. At this point, the “inflammatory phase” begins as plasmin, together with fibrin fragments and TGF-β, triggers the expression of pro-inflammatory proteins and drives the infiltration of inflammatory cells^40^. All of these events result in a burst of inflammation that is directly responsible for the development of radiodermatitis (Fig. 7). In *plg−/−* mice, the fibrin that is accumulated on day 1 after irradiation could not be degraded due to the lack of plasmin, and fibrin fragments therefore could not be formed. Together with the lack of TGF-β induction and activation in *plg−/−* mice, this might explain the lack of an inflammatory response and the absence of radiodermatitis in these mice after irradiation.

TGF-β is a primary factor involved in the tissue damage resulting from irradiation, and several strategies have been tested to target TGF-β signaling as a way to prevent tissue injury^24^, but such treatments have been unsuccessful due to numerous side effects. Our study has for the first time linked plasminogen activation to *TGF-β* expression, suggesting that inhibition of plasminogen might be an upstream event that can be used to suppress TGF-β activation for the prevention of radiodermatitis. Taking into account the necessity of plasmin for the development of radiodermatitis, we propose a novel preventive therapy using clinically approved lysine analogs (such as TXA or ɛ-aminocaproid acid) to inhibit plasminogen activation and thereby prevent or reduce the development of radiodermatitis in cancer patients undergoing radiotherapy. It is possible that the same strategy would also protect internal organs from radiation injury.

The molecular mechanisms for how plasminogen triggers inflammation remain an intriguing question that should be addressed in future studies. Answers to this question might lead to a better understanding of the downstream cascades of plasmin-mediated radiodermatitis, which might in turn lead to the development of novel methods for preventing radiodermatitis in cancer radiotherapy.

## Electronic supplementary material

Supplementary data
