# Supplementary material for: Plasminogen activation is required for the development of radiation-induced dermatitis
Source: Cell Death Dis. 2018 Oct 15;9(11):1051. doi: 10.1038/s41419-018-1106-8 (PMC6189099; doi:10.1038/s41419-018-1106-8)
Supplement: Supplementary file 1 — Supplementary data [file 41419_2018_1106_MOESM1_ESM.pdf]

a

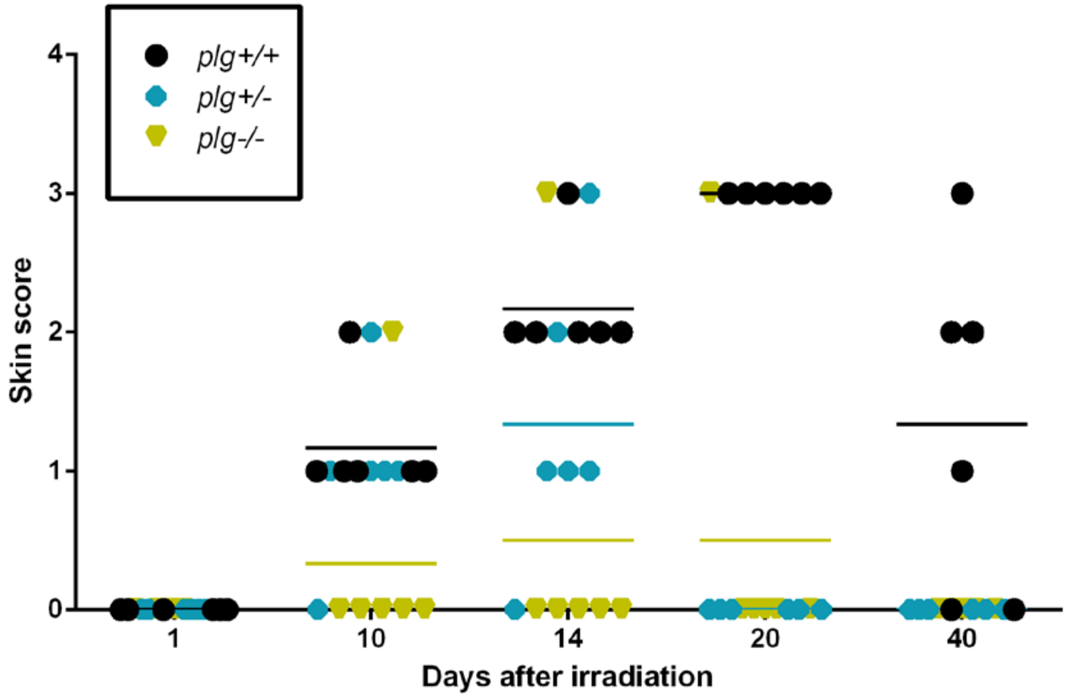

b

	Day 10			Day 14			Day 20			Day 40		
	Plg+/+	*Plg+/-*	*Plg-/-*	*Plg+/+*	*Plg+/-*	*Plg-/-*	*Plg+/+*	*Plg+/-*	*Plg-/-*	*Plg+/+*	*Plg+/-*	*Plg-/-*
Plg+/+	-	*	ns	-	*	ns	-	**	**	-	*	*
Plg+/-	*	-	ns	*	-	ns	**	-	ns	*	-	ns
Plg-/-	ns	ns	-	ns	ns	-	**	ns	-	*	ns	-

Supplementary Fig. 1. A comparison of the quality of dorsal skin in *plg+/+*, *plg+/-* and *plg-/-* mice at different days after irradiation. (a) Skin scores for *plg+/+*, *plg+/-* and *plg-/-* mice at different days after irradiation. (b) Statistical analysis of skin scores between *plg+/+*, *plg+/-* and *plg-/-* mice at different days after irradiation. * $p<0.05$, ns= not significant.

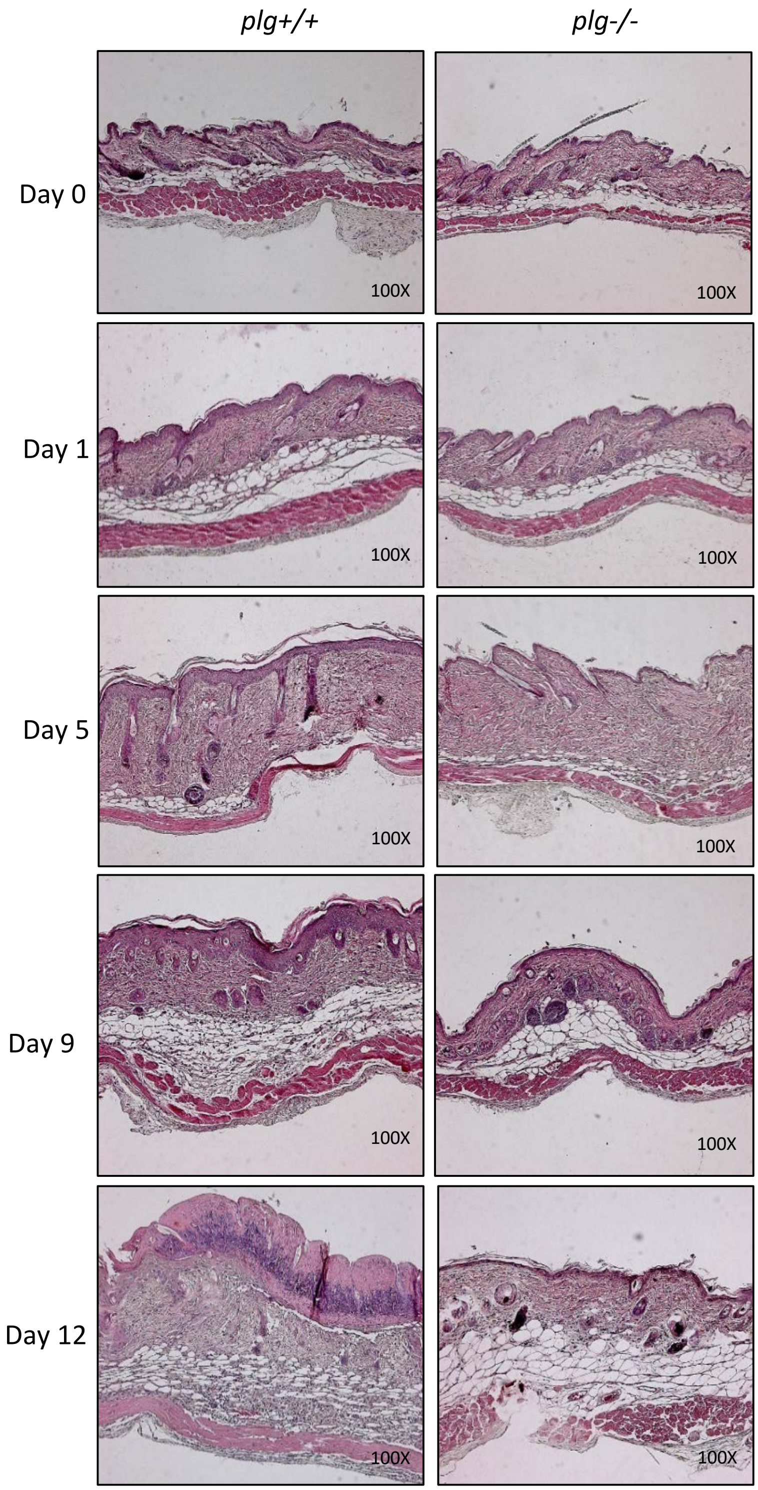

Supplementary Fig. 2. The thickness of epidermis increases significantly after irradiation in *plg+/+* mice, but not in *plg-/-* mice. Representative photographs of H&E stained skin sections from *plg+/+* and *plg-/-* mice at diffrent days after irradiation. Magnification 100x.

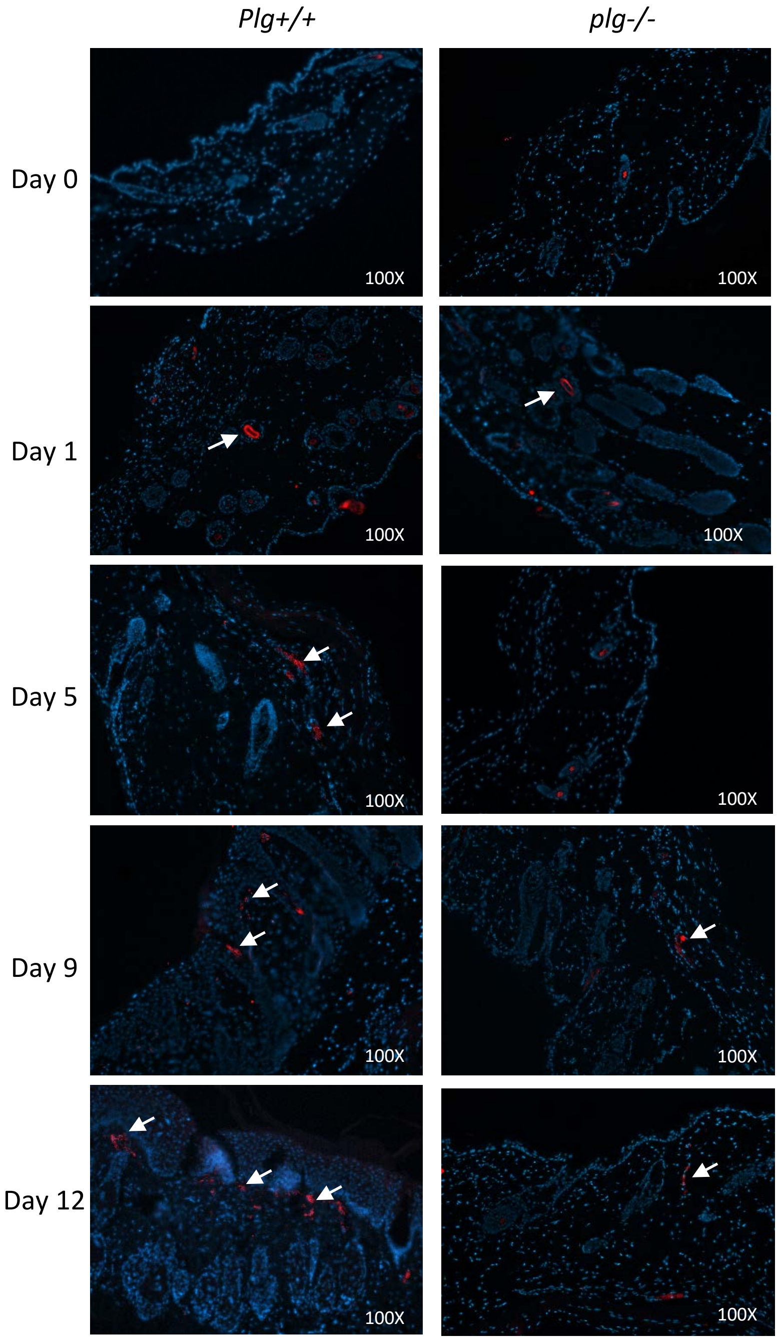

Supplementary Fig. 3. The number of vessels increases significantly after irradiation in *plg*+/+ mice, but not in *plg*-/- mice. Representative photographs of CD31 (red) and DAPI (blue) staining of skin sections from *plg*+/+ and *plg*-/- mice at different days after irradiation. Magnification 100x.

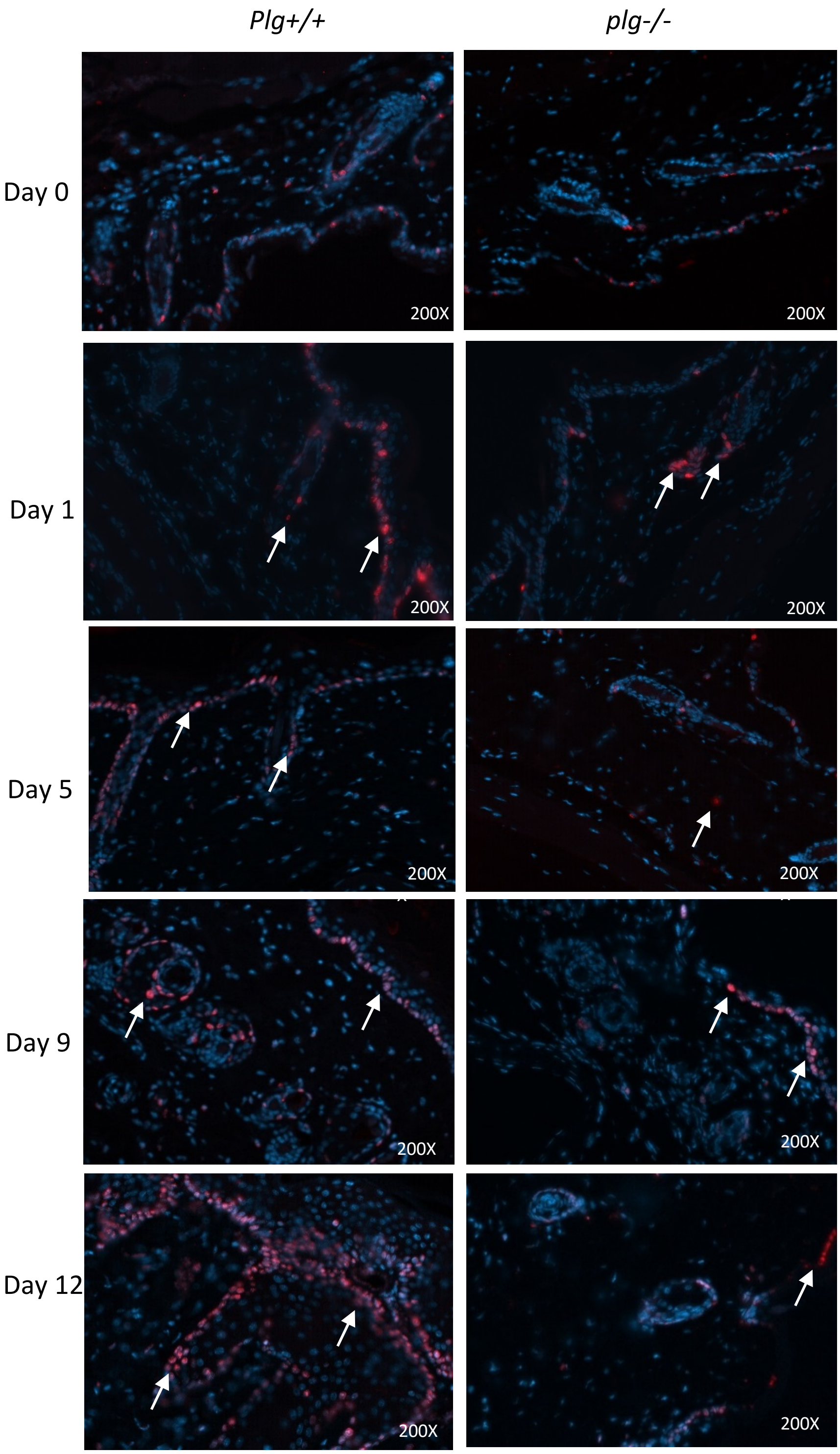

Supplementary Fig. 4. The number of proliferating cells increases significantly after irradiation in *plg*+/+ mice, but not in *plg*-/- mice. Representative photographs of Ki67 (red) and DAPI (blue) staining of skin sections from *plg*+/+ and *plg*-/- mice at different days after irradiation. Magnification 200x.

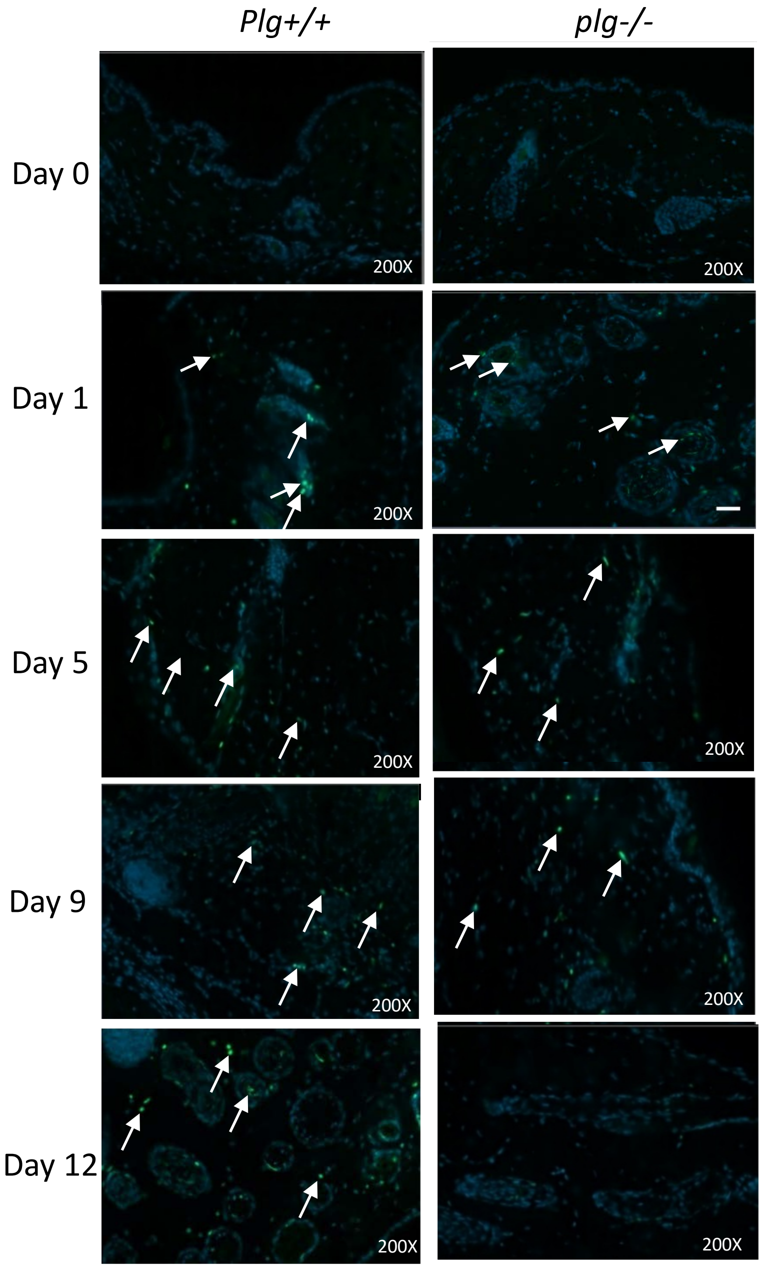

Supplementary Fig. 5. Apoptosis in the skin of *plg*+/+ and *plg*-/- mice after irradiation. Representative photographs of TUNEL (green) and DAPI (blue) staining of skin sections from *plg*+/+ and *plg*-/- mice at different days after irradiation. Magnification 200x.

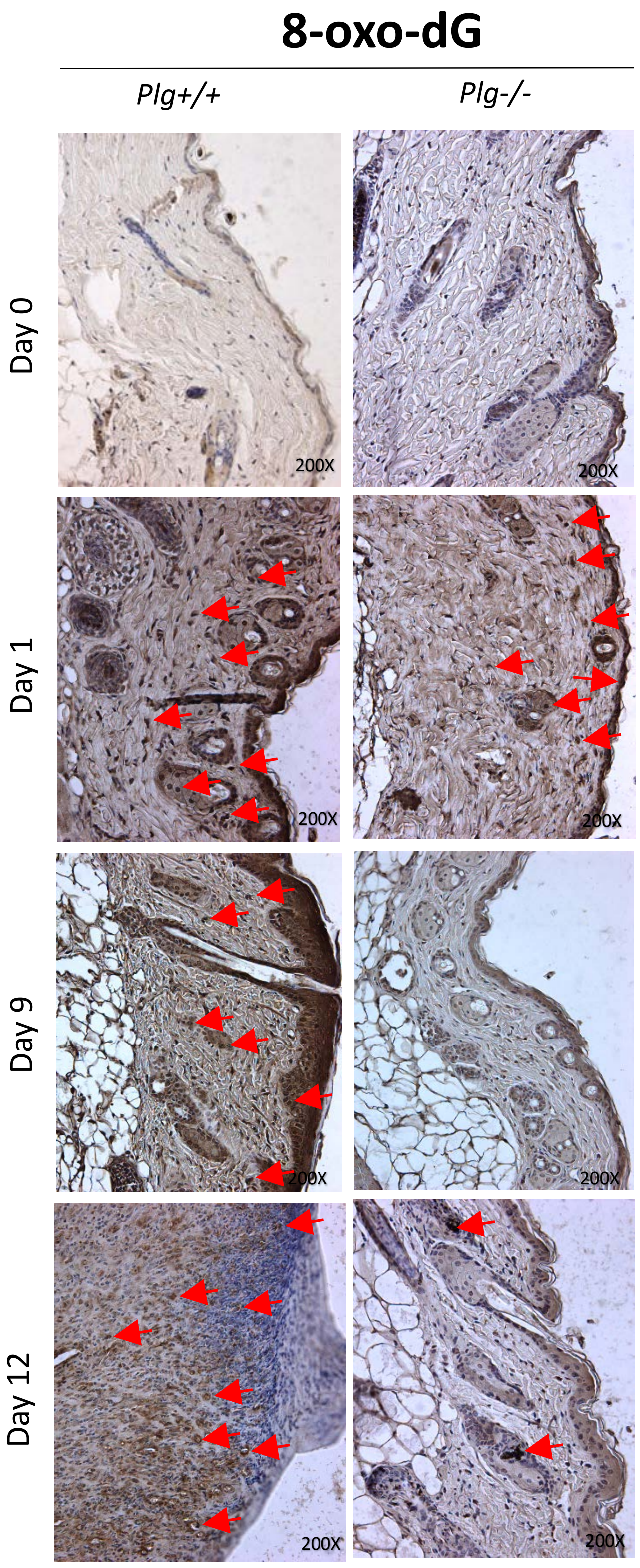

Supplementary Fig. 6. Reactive oxygen spicies (ROS) in the skin of *plg*+/+ and *plg*-/- mice after irradiation. Representative photographs of 8-Oxo-2´-deoxyguanosine (brown) and Hematoxylin (purple) staining of skin sections from *plg*+/+ and *plg*-/- mice at different days after irradiation. Magnification 200x.

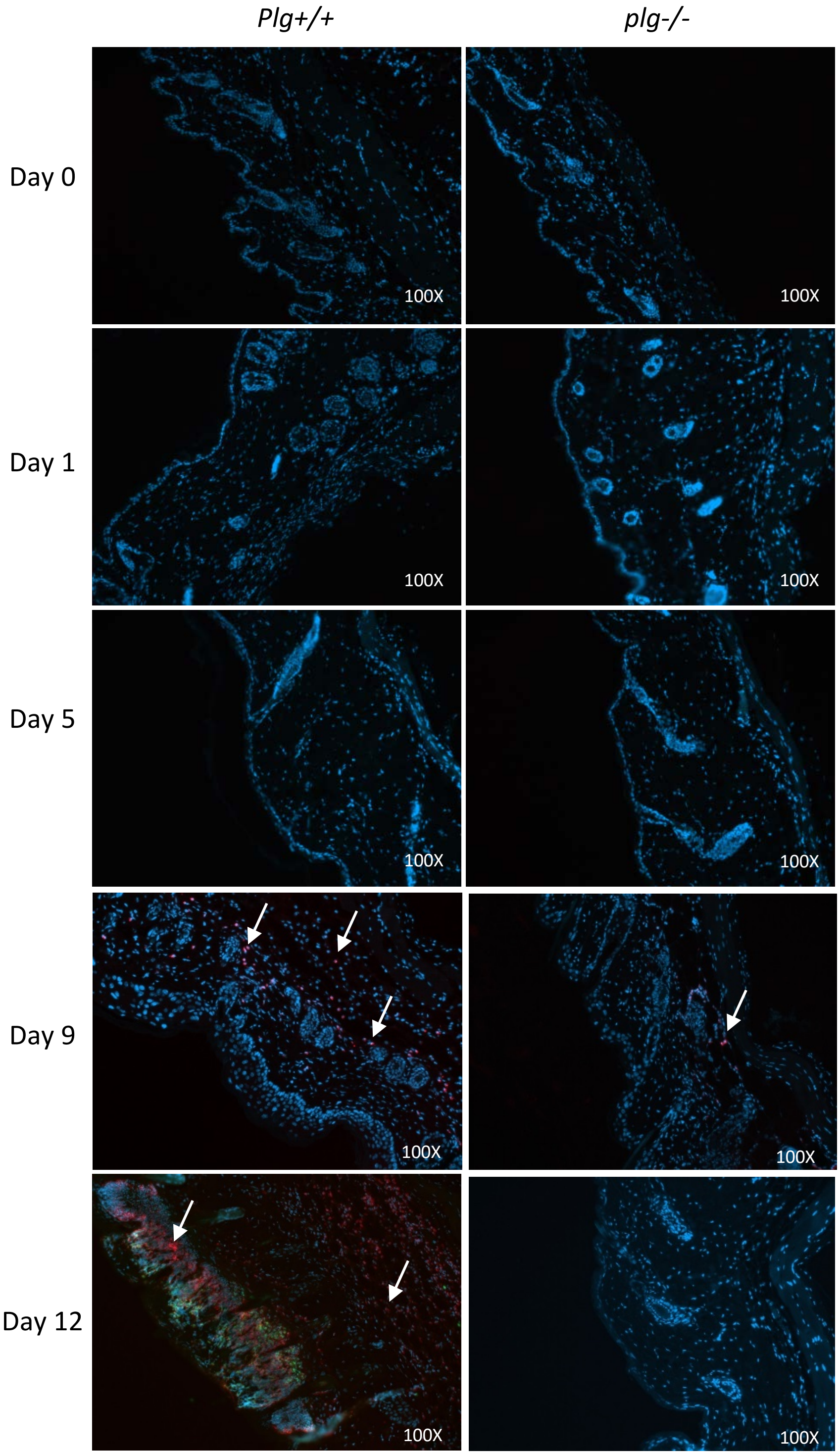

Supplementary Fig. 7. Large numbers of neutrophils and NETS infiltrate irradiated skin in *plg*+/+ mice, but not in *plg*-/- mice. Representative photographs of immunostaining of neutrophils (red) , DAPI (blue), and NETs (citrulinated histon 3, green) on skin sections from *plg*+/+ and *plg*-/- mice at different days after irradiation. Magnification 100x.

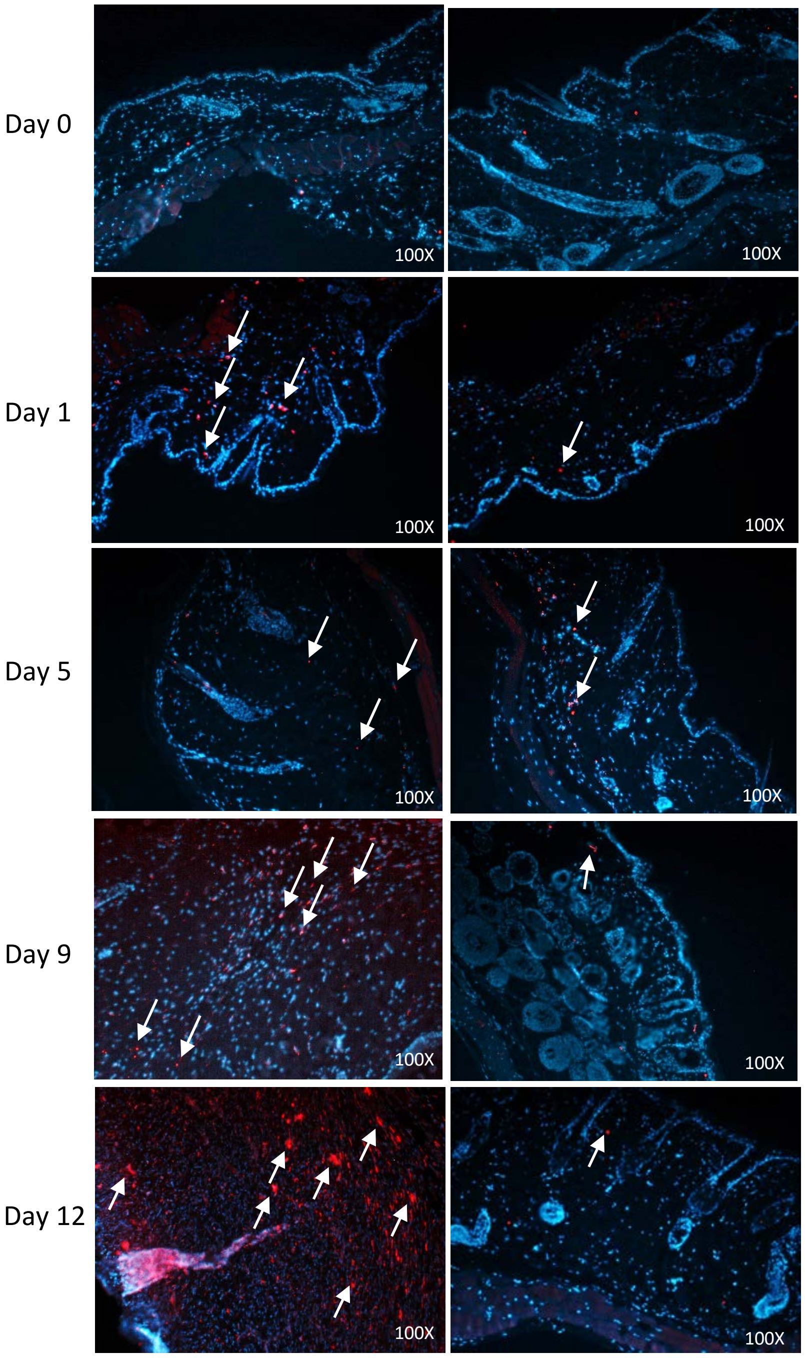

Supplementary Fig. 8. Macrophage accummulation in the skin of *plg*+/+ and *plg*-/- mice after irradiation. Representative photographs of **(**CD68**)** (red) and DAPI (blue) staining of skin sections from *plg*+/+ and *plg*-/- mice at different days after irradiation. Magnification 100x.

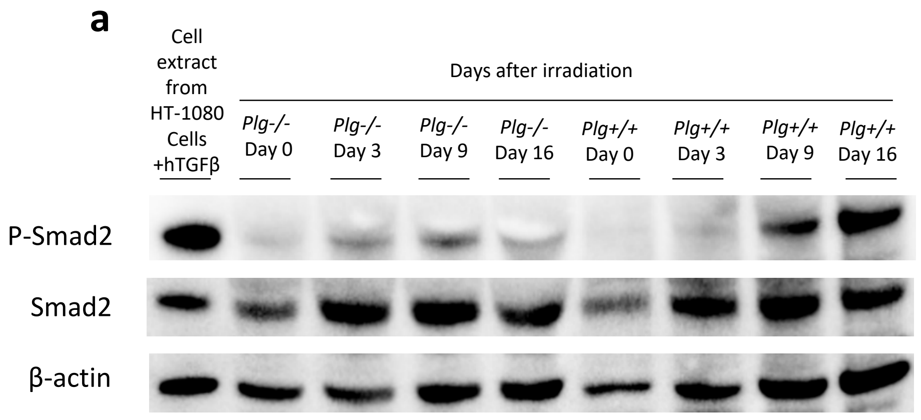

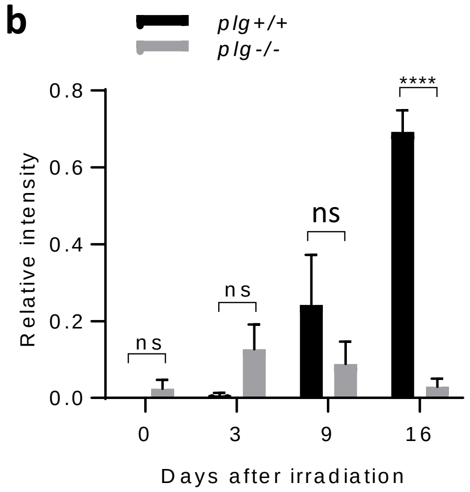

Supplementary Fig. 9. Activation (phosphorylation) of Smad2 in the skin of *plg*+/+ and *plg*-/- mice after irradiation. (a) Representative photograph of western blot of skin samples from *plg*+/+ and *plg*-/- mice at different days after irradiation. β-actin was used as an internal control. HT-1080 cells induced by hTGFβ were used as control for P-Smad2 antibody. (b) Quantification of P-Smad2 from the band intensity (n = 4 per time point).

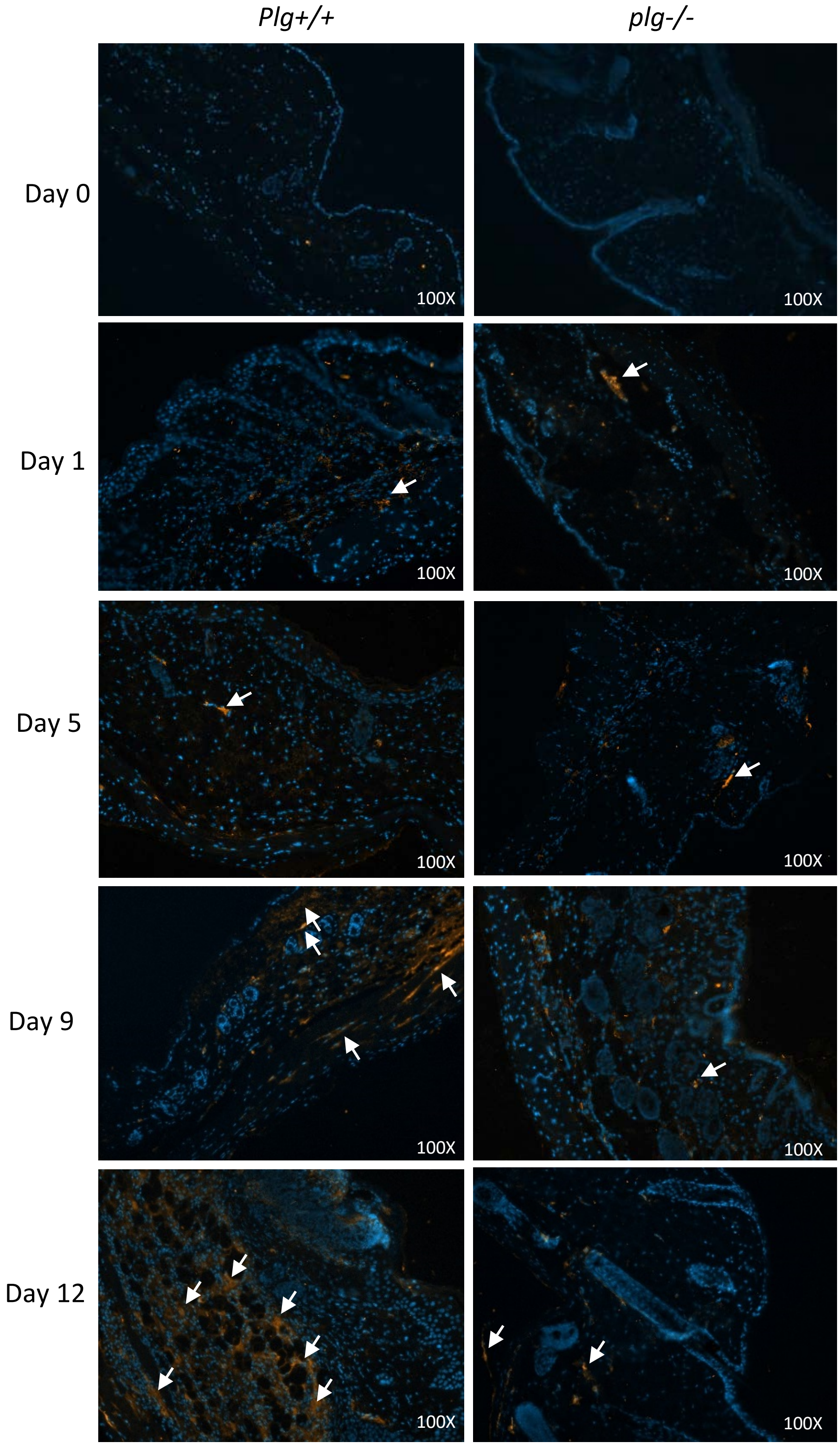

Supplementary Fig. 10. Fibrin accummulation in the skin of *plg*+/+ and *plg*-/- mice after irradiation. Representative photographs of fibrin (orange) and DAPI (blue) staining of skin sections from *plg*+/+ and *plg*-/- mice at different days after irradiation. Magnification 100x.

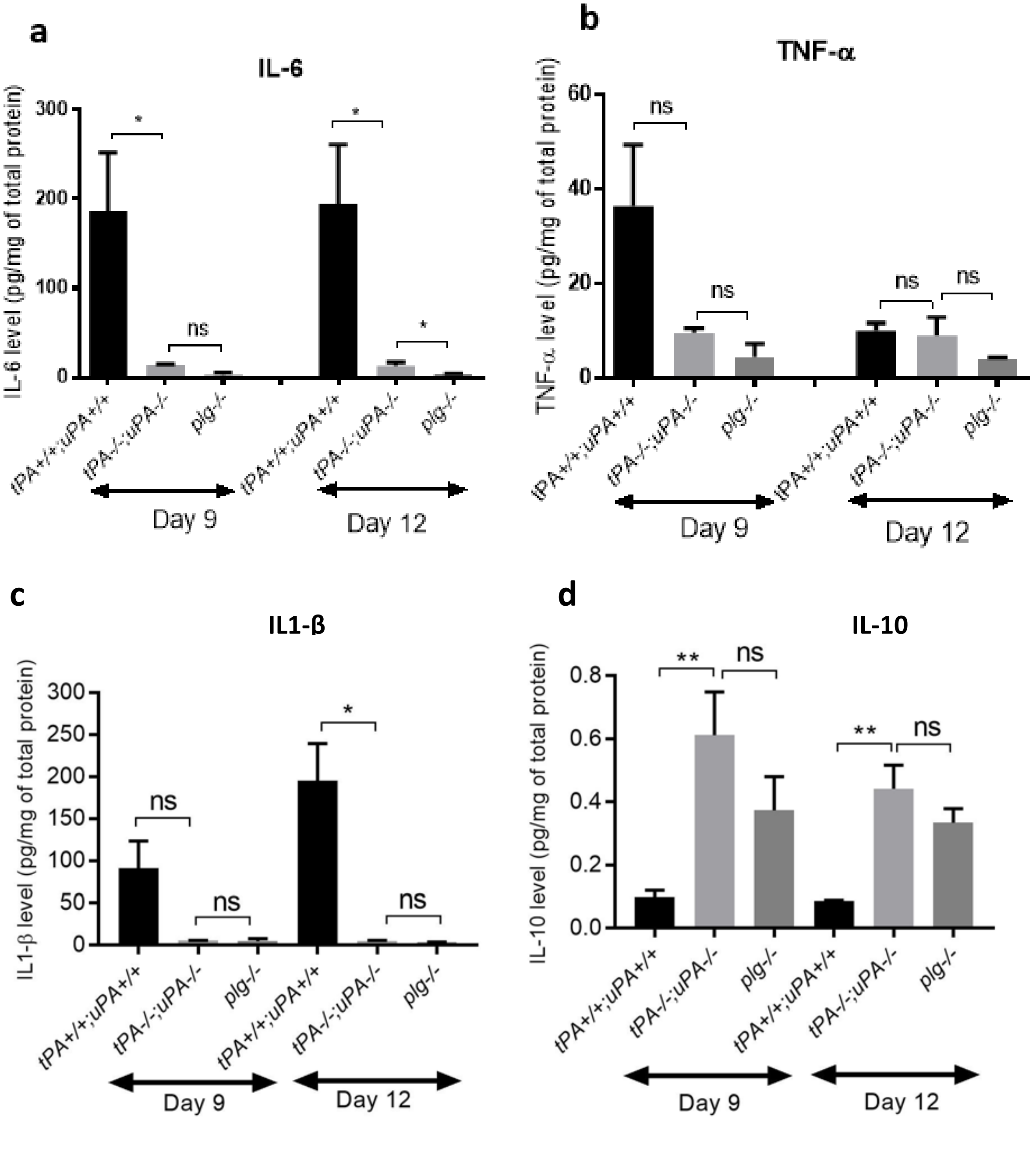

Supplementary Fig. 11. Plasminogen activation is neccessary to induce inflammation after irradiation. Skin samples from WT, *tPA-/-;uPA-/-* and *plg-/-* mice were taken at different times after irradiation. Extracts were prepared and analyzed by ELISA (n ≥ 4 per time point). (a) IL-6 levels , (b) TNF-α levels, (c) IL1-β levels and (d) IL-10 levels in the skin after irradiation.

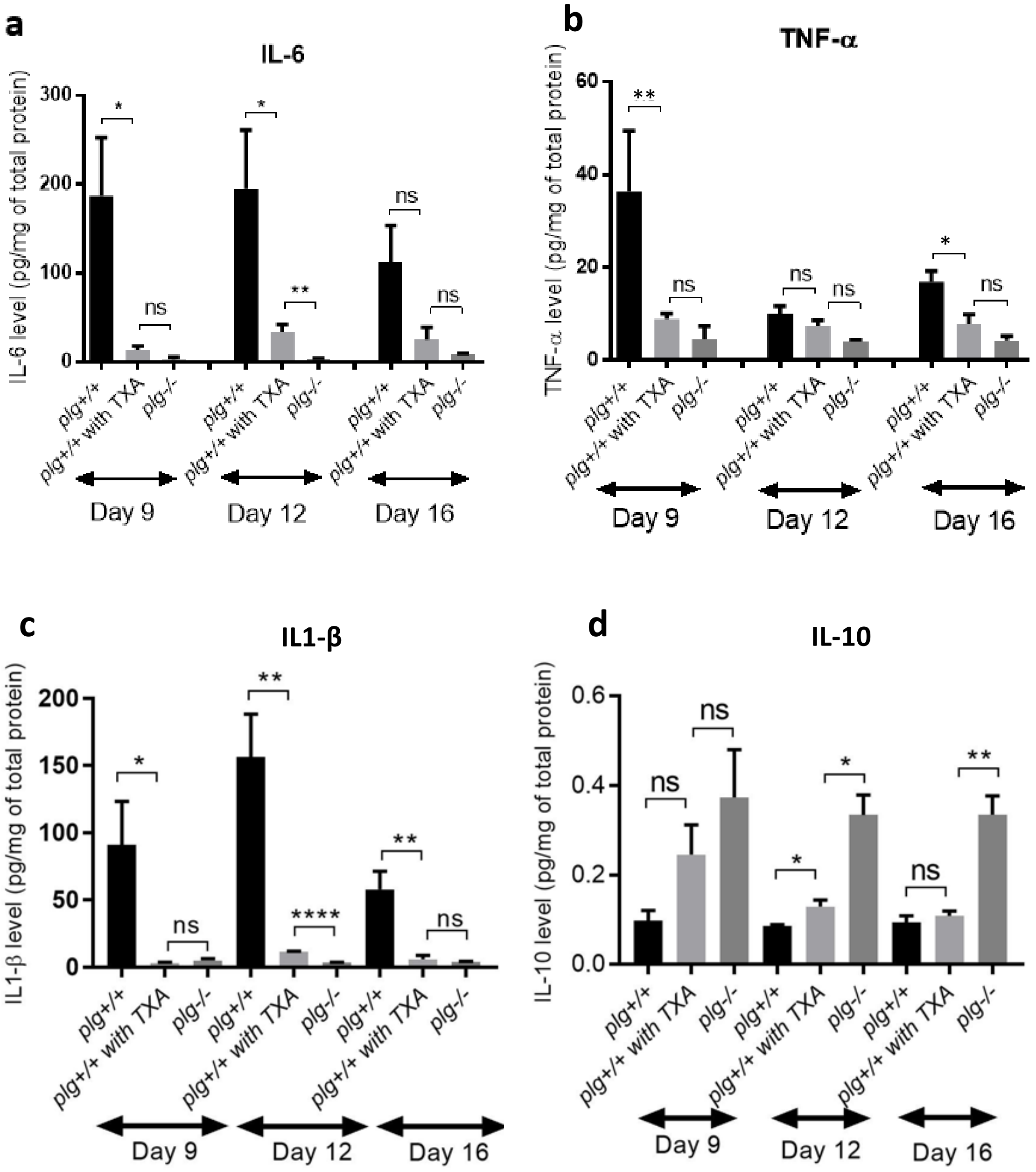

Supplementary Fig. 12. TXA suppresses the accumulation of pro-inflammatory markers in the skin after irradiation. Skin samples from *plg+/+* and *plg-/- mice* and *plg+/+* mice treated with TXA (n ≥ 4) were taken at different times after irradiation and extracts were prepared and analyzed by ELISA (n ≥ 4 per time point) . (a) IL-6 levels, (b) TNF-α levels, (c) IL1-β levels and (d) IL-10 levels in the skin after irradiation.

a

Days after irradiation	Statistical significance between *plg+/+* and and p*lg+/+* with TXA
Day 10	*
Day 14	*
Day 20	ns

b

Days after irradiation	Statistical significance between *plg+/-* and and *plg+/-* with TXA
Day 10	**
Day 14	*
Day 20	ns

Supplementary Table 1. Statistical analysis of skin scores in irradiated mice treated with TXA (Figure 6b and 6d). (a) Statistical analysis of skin scores between *plg+/+* and *plg+/+* treated with TXA. (b) Statistical analysis of skin scores between *plg+/-* and *plg+/-* treated with TXA. * $p<0.05$. ns= not significant.
